# Comparative proteogenomics reveals ecological and evolutionary insights into the organohalide-respiring *Dehalobacter restrictus* strain T

**DOI:** 10.1128/aem.01719-24

**Published:** 2025-03-14

**Authors:** Xiaocui Li, Xiuying Li, Huijuan Jin, Jingjing Wang, Lian Yu, Jun Yan, Yi Yang

**Affiliations:** 1 Key Laboratory of Pollution Ecology and Environmental Engineering, Institute of Applied Ecology, Chinese Academy of Sciences 74763 , Shenyang, Liaoning, China; 2 University of Chinese Academy of Sciences 74519 , Beijing, China; 3 Department of Environmental Engineering, Beijing Institute of Petrochemical Technology 34735 , Beijing, China; 4 CAS Key Laboratory of Forest Ecology and Silviculture, Institute of Applied Ecology, Chinese Academy of Sciences 74763 , Shenyang, Liaoning, China; Georgia Institute of Technology, Atlanta, Georgia, USA

**Keywords:** 1,1,1-trichloroethane, chloroform, reductive dechlorination, organohalide-respiring bacteria, *Dehalobacter*, syntrophy

## Abstract

1,1,1-Trichloroethane (1,1,1-TCA) and chloroform (CF) are persistent groundwater contaminants because of their widespread industrial use as organic solvents and improper disposal in the past. Obligate organohalide-respiring bacteria (OHRB), such as *Dehalobacter* (*Dhb*), play crucial roles in biotransforming and detoxifying natural or anthropogenic halogenated organics including 1,1,1-TCA and CF through reductive dechlorination. Despite their significance, only five *Dhb* strains have been associated with the reductive dechlorination of 1,1,1-TCA or CF. Therefore, cultivating and characterizing novel *Dhb* strains from various environments of different origins worldwide is crucial for understanding the ecology and evolution of *Dhb* and the associated reductive dehalogenase (RDase) genes. This study reports the enrichment and investigation of a novel *Dhb* population capable of reducing 1,1,1-TCA to 1,1-dichloroethane, CF to dichloromethane, 1,1,2-TCA to vinyl chloride/1,2-dichloroethane, and 1,2,4-trichlorobenzene to 1,2-dichlorobenzene. The capability for dechlorinating both aliphatic and aromatic compounds was observed for the first time in the sediment sourced from the Xi River situated in the North China Plain. Comparative genomic analysis of *Dhb* strains revealed genome contraction might have resulted in the loss of various gene family members, contributing to the syntrophy interactions (e.g., cobalamin, hydrogen, and acetate) of *Dhb* with other anaerobes (e.g., fermenters and acetogens). Proteogenomic and phylogenetic analysis confirmed the highly expressed 1,1,1-TCA/CF-dechlorinating RDase, designated as TcaA, shared 94.7–96.7% amino acid sequence similarities with RDases, such as ThmA, CfrA, and TmrA. This study expands knowledge on *Dhb* biogeography and evolution while providing insights into potential syntrophy interactions supporting organohalide respiration by *Dhb*. The findings have implications for developing the novel biotechnologies for the remediation of halogenated alkane-contaminated sites.

**IMPORTANCE:**

Organohalide-respiring bacteria (OHRB) are essential for breaking down harmful pollutants in the environment. This study investigates a newly discovered OHRB capable of degrading multiple contaminants, including persistent 1,1,1-trichloroethane and chloroform. By understanding its unique abilities and interactions with other microbes, we gain valuable insights into how these bacteria evolve and function, enabling the development of improved bioremediation strategies to clean up polluted sites.

## INTRODUCTION

Under anoxic conditions, halogenated organics are commonly biotransformed by organohalide-respiring bacteria (OHRB) through microbial reductive dechlorination (1). These bacteria can use halogenated organic compounds as electron acceptors and hydrogen (H_2_) or organic acids (e.g., formate and acetate) as electron donors for reductive dehalogenation, a process that conserves energy for growth via electron transport phosphorylation (2). Obligate OHRB (e.g., *Dehalococcoides* [*Dhc*], *Dehalogenimonas* [*Dhgm*], and *Dehalobacter* [*Dhb*]) have received the most attention because of their dedication to detoxifying prioritized halogenated pollutants (3), their demonstrated relevance for biogeochemical cycling of carbon and halogen elements (4), and their wide applications (5) for *in situ* bioremediation. Similar to *Dhc* and *Dhgm*, *Dhb* can also respire on a wide spectrum of halogenated organic compounds, including chloroethenes (e.g., tetrachloroethene [PCE]) (6), chloroethanes (e.g., 1,1,1-trichloroethane [1,1,1-TCA] and 1,1,2-TCA) (7), chloromethanes (e.g., chloroform [CF]) (8), chlorobenzenes (e.g., 1,2-dichlorobenzene [1,2-DCB]) (9), and other halogenated compounds (e.g., hexachlorocyclohexane and tetrachlorophthalide) (10, 11). Among these halogenated organics, 1,1,1-TCA and CF are particularly challenging due to their extensive chlorination and the inherent strength of their carbon-chlorine bonds. These characteristics result in chemical stability and high recalcitrance, necessitating the specialized microorganisms equipped with the specific enzymes, such as reductive dehalogenase (RDase), for their breakdown. Additionally, 1,1,1-TCA and CF can be toxic to many microorganisms, further limiting the number of species capable of degrading these compounds. For instance, 1,1,1-TCA and CF have been found to severely disrupt various biological processes, including methanogenesis (12, 13) and the reductive dechlorination of chloroethenes (14, 15). Therefore, it is of paramount importance to identify the microbial solutions to mitigate their effects on different ecosystems, for the benefit of human and ecological health.

Currently, only *Dhb* strains (e.g., strains CF, TCA1, UNSWDHB, THM1, and 8M) and *Desulfitobacterium* sp. strain PR have been demonstrated to dechlorinate 1,1,1-TCA or CF (7, 16–21), and neither *Dhc* nor *Dhgm* can respire and detoxify 1,1,1-TCA or CF. *Dhb* sp. strain TCA1 was the first OHRB isolated with the capacity to reductively dechlorinate 1,1,1-TCA to 1,1-dichloroethane (1,1-DCA) and ultimately to chloroethane (CA) (16). Subsequent studies identified *Dhb* sp. strains CF and DCA from the enrichment culture ACT-3, which demonstrates the distinct dechlorination capabilities: strain CF dechlorinates CF and 1,1,1-TCA but not 1,1-DCA, whereas strain DCA dechlorinates 1,1-DCA but not CF or 1,1,1-TCA (17, 21, 22). *Desulfitobacterium* sp. strain PR was found to dechlorinate 1,1,1-TCA to CA via 1,1-DCA and could also transform CF to chloromethane (CM) via dichloromethane (DCM) (18). While *Dhb* sp. strain UNSWDHB exhibits high CF tolerance (e.g., up to 4 mM), it slowly dechlorinates 1,1,1-TCA and 1,1-DCA and incompletely removes them with minimum threshold concentrations of 0.12 and 0.07 mM, respectively (7). *Dhb* sp. strain THM1, the first known OHRB to couple growth with reductive debromination of bromoform, can also dechlorinate CF and 1,1,1-TCA (19). The *Dhb* strain 8M-containing culture is capable of dechlorinating CF to DCM completely, but the conversion of 1,1,1-TCA to 1,1-DCA, 1,1-DCA to CA, and bromoform to dibromomethane was incomplete (20). Importantly, none of the strains discussed above have demonstrated the ability to dechlorinate halogenated aromatic compounds. No OHRB strain has yet been discovered that can completely dechlorinate 1,1,1-TCA to an innocuous end product (e.g., ethane). Additionally, five RDases, such as CtrA, TmrA, DcrA, CfrA, and ThmA, have been identified and characterized from the abovementioned strains catalyzing 1,1,1-TCA and/or 1,1-DCA dechlorination (Table S1).

While there have been increasing studies on OHRB and their roles in the dechlorination of halogenated organic compounds, the knowledge regarding the diversity and capabilities of OHRB, especially those related to the degradation of 1,1,1-TCA and CF, remains limited. Only a few *Dhb* strains have been shown to dechlorinate these compounds, and there is still a lack of understanding of the ecological and evolutionary aspects of these bacteria in different environments. The acquisition of an increased number of OHRB isolates from diverse global environments is crucial for gaining insights into the ecological and evolutionary aspects of OHRB. The discovery of novel OHRB not only adds to our understanding of microbial biogeography but also contributes to the bioremediation and cleanup of the sites contaminated with halogenated pollutants. In this study, we present the first demonstration of the reductive dechlorination of 1,1,1-TCA to 1,1-DCA in the microcosms using the sediment from the Xi River in the North China Plain. A *Dhb* population was enriched and found capable of dechlorinating both chlorinated aliphatic and aromatic compounds, such as 1,1,1-TCA, CF, 1,1,2-TCA, and 1,2,4-trichlorobenzene (1,2,4-TCB). *Dhb* was the dominant genus in the 1,1,1-TCA-dechlorinating cultures, with growth coupled to dechlorination (7.53 ± 0.38 × 10⁷ cells/µmole chloride released). Metagenomic analysis of a draft *Dhb* genome identified a 1,1,1-TCA RDase (TcaA) with high similarity to known 1,1,1-TCA RDases, expanding our knowledge of OHRB diversity.

## MATERIALS AND METHODS

### Microcosm setup and enrichment

The sediment samples were collected from the Xi River (41.6628°N, 123.1055°E, Shenyang, Liaoning, China), which is contaminated with various halogenated hydrocarbons and heavy metals (23). Microcosms were established inside an anoxic chamber (Coy Laboratory Products, Ann Arbor, MI, USA) by dispensing ~5 g sediment slurry to 120 mL serum bottles filled with a headspace of N_2_/CO_2_ (80/20, vol/vol) and 80 mL of bicarbonate-buffered mineral salt medium supplemented with 5 mM lactate and the Wolin vitamin mix with 50 µg L^−1^ vitamin B_12_ (24). Acetate (carbon source) and H_2_ (electron donor) can be generated from the catabolism of lactate (25). The serum bottles were sealed with butyl rubber stoppers (Shenzhen Fushiyuan Rubber & Plastic Factory, Guangdong, China) and secured with aluminum crimp caps (Ningbo Hoonpo Laboratory Technology Co., Ltd., Zhejiang, China). Neat 1,1,1-TCA (*ca*. 6 µL or 59.36 µmol, 0.52 mM aqueous concentration) and H_2_ (~10 mL/bottle) were added as electron acceptor and electron donor, respectively. Following the dechlorination of 1,1,1-TCA to 1,1-DCA, continuous transfers (3.75%, vol/vol) to the fresh mineral salt medium (24) yielded the solid-free 1,1,1-TCA-dechlorinating enrichment cultures. Autoclaved cultures amended with 1,1,1-TCA served as abiotic controls. All serum bottles were incubated at 30°C in the dark without shaking. The microcosms and transferred enrichment cultures were set up in triplicate. The potential of the solid-free enrichment cultures to reductively dechlorinate CF, 1,2-DCA, 1,1,2-TCA, PCE, trichloroethylene (TCE), and various chlorinated phenols (see the supplementary information) was determined in 120 mL serum bottles, each containing 80 mL basal medium.

### DNA extraction and 16S rRNA gene amplicon sequencing

Following the complete dechlorination of 1,1,1-TCA, cells of the microcosms, as well as from the second- and sixth-generation cultures, were collected by filtering 1 mL of each liquid culture onto 0.22 µm MCE filters (Jinteng, Tianjin, China). Genomic DNA was extracted from the filter using a TIANamp soil DNA kit (Tiangen Biotech Co., Ltd., Beijing, China) following the manufacturer’s instructions. The V3-V4 regions of bacterial and archaeal 16S rRNA genes were amplified using the primer sets V3-V4-F and V3-V4-R (Table S2). Libraries were constructed using the MetaVx Library Construction Kit (GENEWIZ, USA). Paired-end sequencing targeting 16S rRNA genes was performed using the Illumina MiSeq PE250/300 platform according to established protocols (26).

### Metagenome sequencing, assembly, and binning

DNA from the tenth-generation culture was extracted using a CTAB protocol recommended by the Joint Genome Institute of the US Department of Energy (JGI, Walnut Creek, CA, USA) (17). The library was constructed using a NovaSeq 6000 S4 kit. Metagenomic sequencing was performed on a NovaSeq 6000 sequencer (Illumina, Inc., San Diego, CA, USA). Raw sequence data were processed with Readfq (V8, https://github.com/cjfields/readfq) to acquire the filtered sequence data for subsequent analysis. After being trimmed and filtered, the resulting 35,338,878 paired-end reads were assembled using the JGI Metagenome Assembly Pipeline (https://github.com/kbaseapps/jgi_mg_assembly). Metagenomic short-read profiling and taxonomic classification were performed using Kaiju v1.7.3 (27). Metagenomic contigs were classified with Maxbin2 v2.2.4 (28). Metagenome-assembled genomes (MAGs) were assessed with CheckM (29) for completeness and contamination of bacterial or archaeal genomes. The taxonomic affiliation was performed with the GTDB-Tk software toolkit (30) and the Type (Strain) Genome Server (TYGS, https://tygs.dsmz.de) (31). High-quality MAGs included the draft genome sequence of one *Dhb* strain, designated as strain T. The draft genomes of strain T and other non-dechlorinating populations were annotated using PATRIC version 3.6.12 with default parameters (32).

### Proteomics analysis

After the complete dechlorination of 1,1,1-TCA and CF, the biomass from 640 mL enrichment cultures was harvested by centrifugation for protein identification. Trypsin-digested peptides were desalted, concentrated, and analyzed using LC-MS/MS on a Q Exactive HF mass spectrometer. MS data were searched against the protein database of strain T using MaxQuant. Identified proteins had a false discovery rate ≤0.01 after filtering peptide-spectrum matches at ≤0.01 FDR. Detailed methods and procedures are described in the supplementary information.

## RESULTS

### Reductive dechlorination in sediment microcosms and enrichment cultures

**Fig 1:**
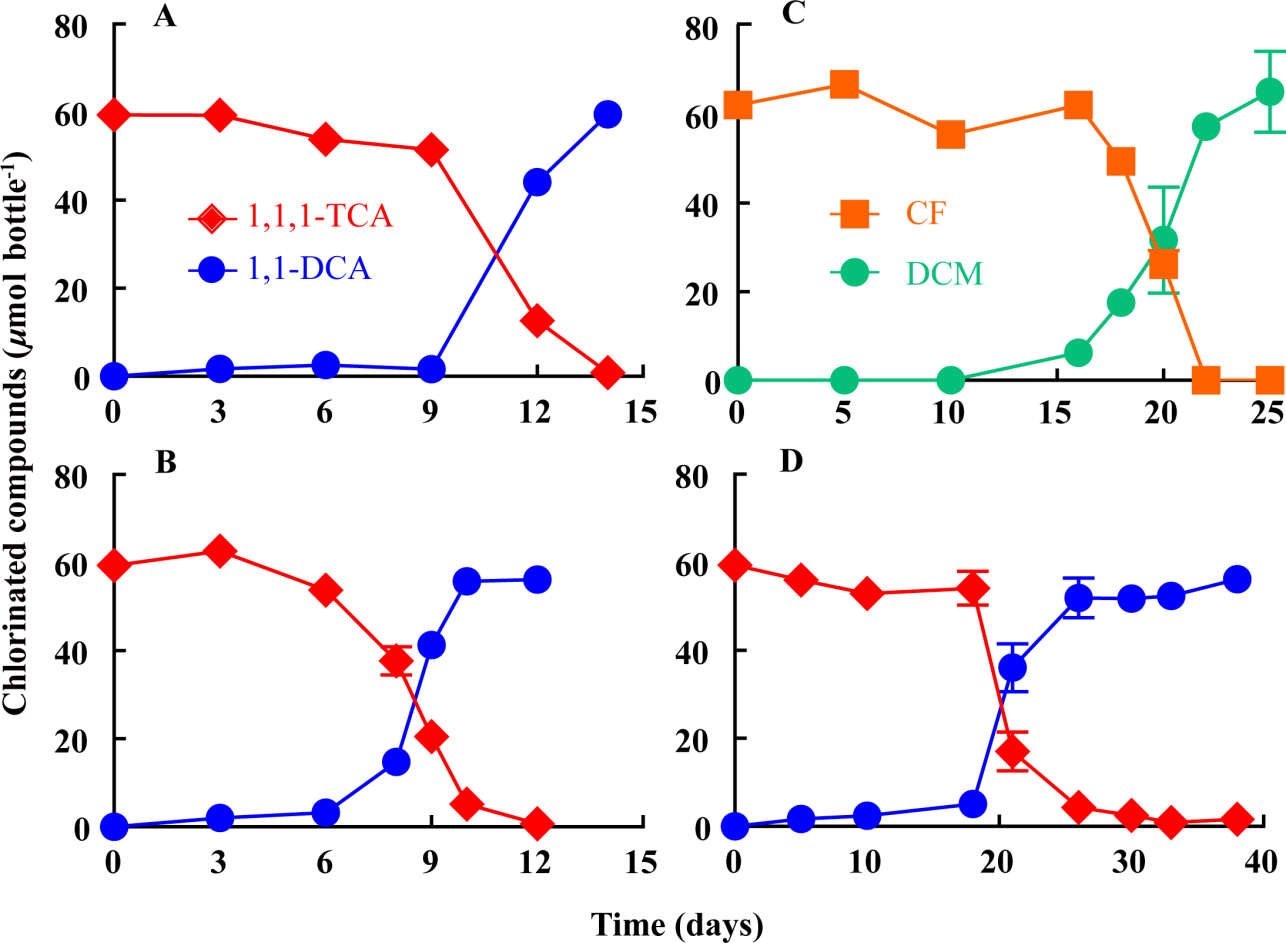
Reductive dechlorination of 1,1,1-TCA or CF in enrichment cultures. (**A, B**) Dechlorination of 1,1,1-TCA to 1,1-DCA in the sixth- and twelfth-generation cultures amended with lactate, respectively. (**C**) Dechlorination of CF to DCM in the sixth-generation culture amended with lactate. (**D**) Dechlorination of 1,1,1-TCA to 1,1-DCA in the sixth-generation culture amended with acetate. The error bars represent standard deviations of triplicate bottles and are not displayed when smaller than the symbol.

Anaerobic microcosms dechlorinated ~60 µmol 1,1,1-TCA (~0.52 mM) predominantly to 1,1-DCA within 60 days of incubation, with methane and by-product 1,1-dichloroethylene (1,1-DCE), vinyl chloride (VC) and ethene detected. Following continuous transfers, the enrichment cultures amended with lactate and H_2_ maintained the ability to dechlorinate ~60 µmol 1,1,1-TCA to 1,1-DCA within 2 weeks with an accelerated dechlorination rate of 65 µM day^−1^ (Fig. 1A and B), concomitantly producing minimal 1,1-DCE (<0.2 µmol), but methane, VC, and ethene were not detected. In autoclaved microcosms, less than 2% of 1,1,1-TCA was abiotically transformed to 1,1-DCE, and no 1,1-DCA was detected after 4 months’ monitoring (Fig. S1), suggesting the biotic reaction leading to the conversion of 1,1,1-TCA to 1,1-DCA. The solid-free enrichment cultures could also dechlorinate ~62 µmol CF (~0.78 mM) to DCM within 22 days (Fig. 1C) and dechlorinate 1,1,2-TCA to 1,2-DCA and VC at a ratio close to 1:1 during an extended monitoring period (i.e., ~130 days) (Fig. S2). However, chlorinated compounds, such as PCE, TCE, 1,2-DCA, and chlorophenols, could not be dechlorinated by enrichment cultures within a 160-day monitoring period. Compared with vitamin B_12_-amended, the average dechlorination rate of 1,1,1-TCA was relatively slow in the enrichment culture without the addition of vitamin B_12_ (Fig. S3), indicating the necessity of vitamin B_12_ for 1,1,1-TCA reductive dechlorination. The enrichment cultures amended with acetate and H_2_ took over 1 month to transform ~60 µmol 1,1,1-TCA to 1,1-DCA with an average dechlorination rate of 14 µM day^−1^ (Fig. 1D). In all enrichment cultures examined, there was a consistent delay of approximately 1 week in the dechlorination of 1,1,1-TCA, which has been also observed in previous studies (33).

### Microbial community structures

**Fig 2:**
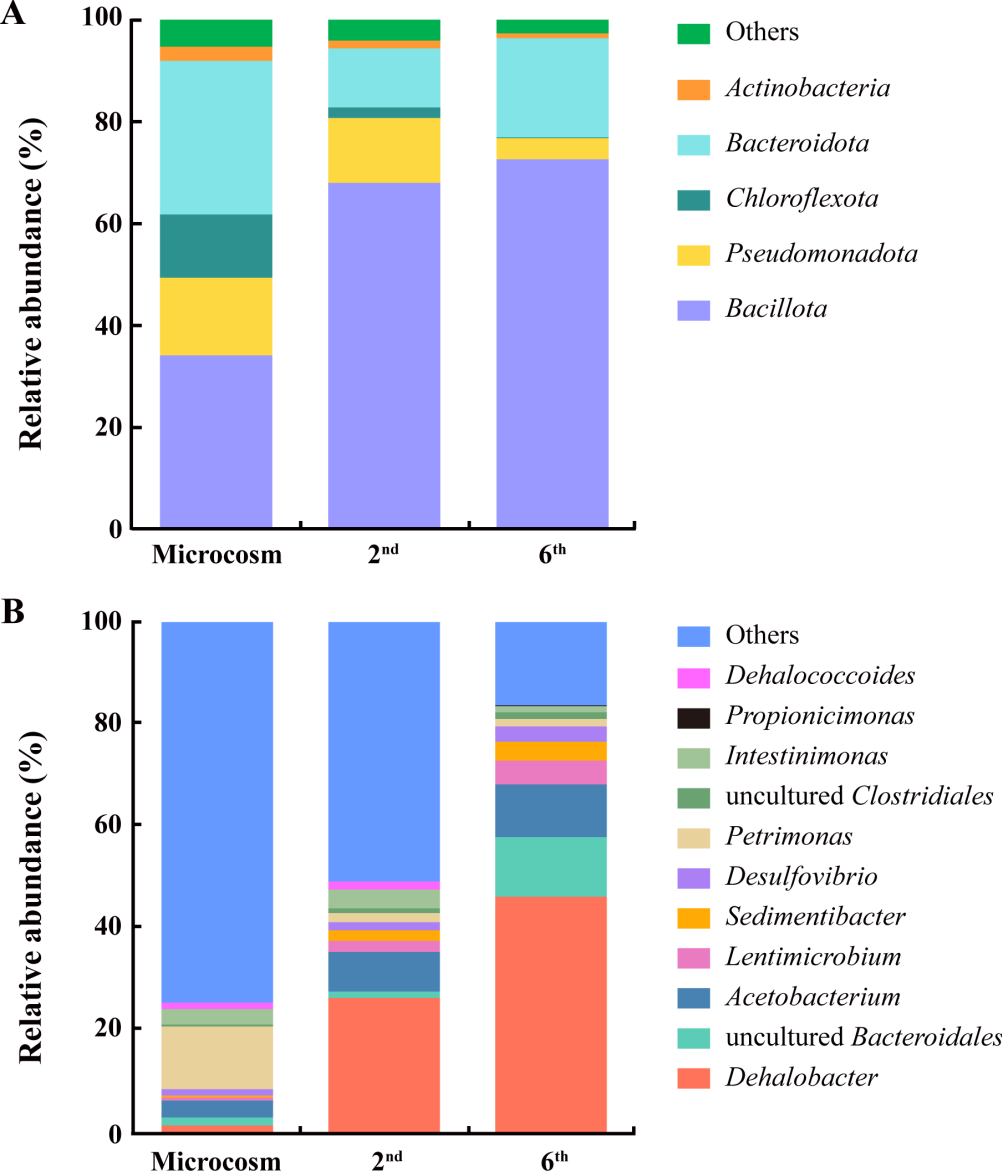
Microbial community compositions at the phylum level (**A**) and genus level (**B**) in the sediment microcosm and the second- and sixth-generation cultures following complete conversion of 1,1,1-TCA to 1,1-DCA, respectively. Note that “Others” refers to the sum of OTUs affiliated with low-abundance phyla and genera (≤1% of the total sequences).

Polymerase chain reaction (PCR) assay targeting 16S rRNA genes of dechlorinating bacteria combined with Sanger sequencing of the PCR amplicon unraveled the presence of a *Dhb* population (Fig. S4A), indicating that *Dhb* may contribute to 1,1,1-TCA dechlorination. To further identify the population(s) responsible for 1,1,1-TCA-to-1,1-DCA dechlorination, 16S rRNA gene amplicon sequencing was applied to profile the community structures of the microcosms and the second- and sixth-generation cultures. The dominant phyla were *Bacillota*, *Pseudomonadota*, *Bacteroidota*, and *Chloroflexota*, which together represented 91.9–96.4% of the total sequences in the sediment microcosms, the second- and sixth-generation cultures (Fig. 2A). *Bacillota* (33.8–72.5%) and *Bacteroidota* (11.6–30.3%) remained the most abundant phyla in the enrichment cultures, while the relative abundances of *Pseudomonadota* and *Chloroflexota* sequences decreased from 15.3% and 12.5% in the microcosms to 4.2% and 0.2% in the sixth-generation cultures, respectively. At the genus level (Fig. 2B), the most abundant OTUs in the microcosms belong to *Petrimonas* (12.2%) within the *Bacteroidota* phylum, but *Petrimonas* sequences were detected at a relatively low abundance (<1.4%) in the sixth-generation culture. *Dhb* sequences, comprising only 1.8% of the total sequences in the microcosms, but after consecutive transfers, became dominant and represented 26.7% and 46.5% of the total sequences in the second- and sixth-generation cultures, respectively (Fig. 2B). In addition to *Dhb*, relative abundances of *Bacteroidales*, *Acetobacterium*, *Lentimicrobium*, *Desulfovibrio*, and *Sedimentibacter* were above 1.0% in the sixth-generation culture, and the total relative abundances of *Dhb* together with these five genera reached 80.0%. *Dhc* was also detected in the microcosms and the second-generation culture, albeit at relatively low abundances (<1.6%); nonetheless, *Dhc* was lost after six consecutive transfers (Fig. 2B). Overall, the significant increase in *Dhb* abundance following continuous transfers suggested the contribution of *Dhb* to the dechlorination of 1,1,1-TCA.

### *Dhb* growth coupled with reductive dechlorination

**Fig 3:**
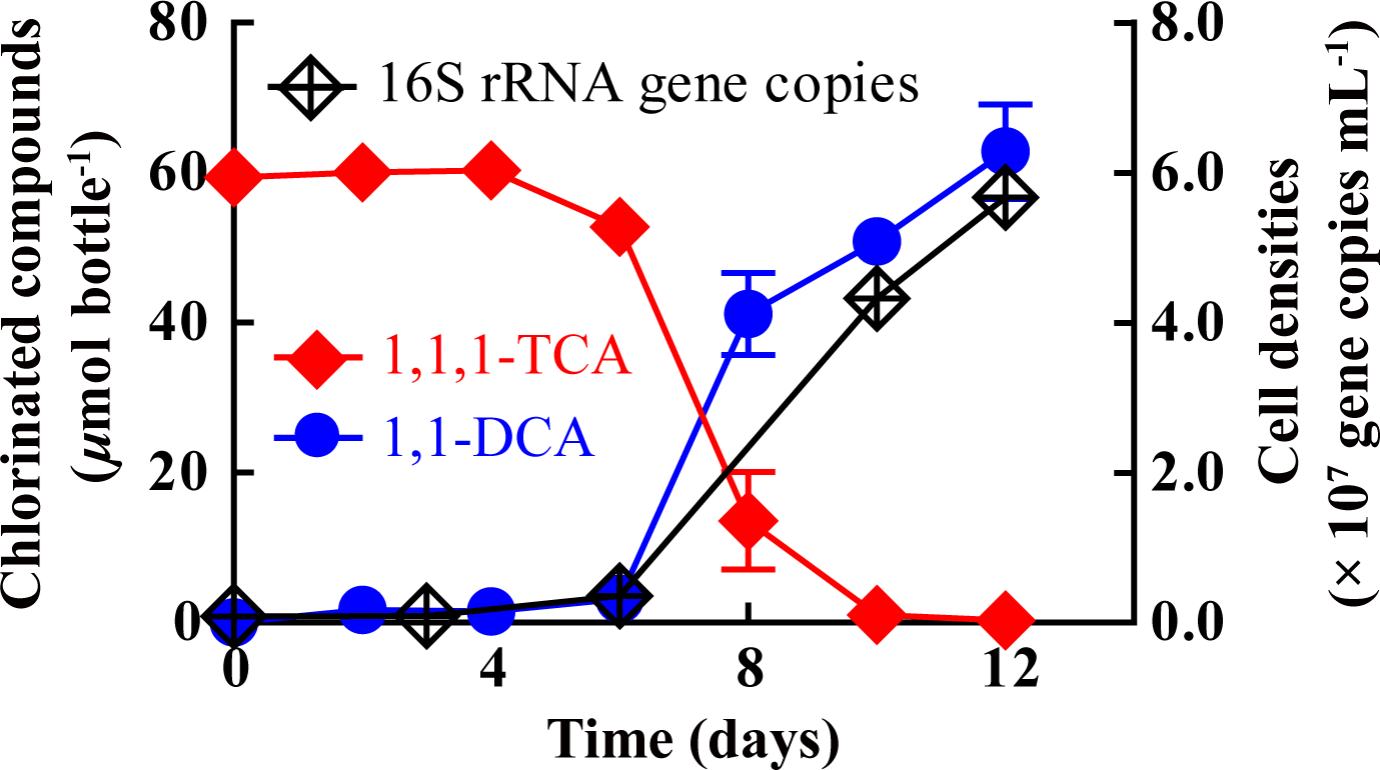
Reductive dechlorination of 1,1,1-TCA to 1,1-DCA and growth of strain T in the thirteenth-generation culture. Cell number estimates were calculated under the assumption that strain T’s genome contains one copy of the 16S rRNA gene. The error bars represent standard deviations of triplicate bottles and are not displayed when smaller than the symbol.

In enrichment cultures, the dechlorination of 1,1,1-TCA to 1,1-DCA began after a lag phase of 6–10 days, during which no growth of *Dhb* was observed (Fig. 3). Following the consumption of the initial amount of 1,1,1-TCA, the number of *Dhb* 16S rRNA gene copies per mL increased from initial 8.28 ± 0.69 × 10^5^ to 5.67 ± 0.29 × 10^7^ (a 68.4-fold increase) in the thirteenth-generation culture (Fig. 3), achieving an average yield of 7.53 ± 0.38 × 10^7^ cells per µmole of chlorine released during the stoichiometric transformation of ~60 µmol 1,1,1-TCA to 1,1-DCA (Table 1). However, negligible increases in *Dhb* cell density were observed in cultures that did not receive 1,1,1-TCA amendment (Table 1). These results suggested that the growth of the *Dhb* population may be coupled with the dechlorination of 1,1,1-TCA to 1,1-DCA. We further applied the qPCR assay to determine whether the dechlorination of other chlorinated organic compounds could support *Dhb* growth and found that the *Dhb* growth yields during the dechlorination of CF to DCM and 1,1,2-TCA to 1,2-DCA/VC were 1.41 ± 0.33 × 10^8^ and 3.02 ± 0.01 × 10^7^ cells per µmole of chlorine released, respectively (Table 1), which are generally comparable to other examined *Dhb* strains, as shown in Table S1.

**TABLE 1:** Growth yields of strain T in enrichment cultures amended with 1,1,1-TCA and other organochloride compounds

Dechlorination process	*Dhb* cell densities (cells mL^−1^)*^a^*		Fold increase^*c*^	Growth yield^*d*^ (cells per μmol Cl^−1^ released)
	Initial	Final^*b*^		
1,1,1-TCA^*e*^→1,1-DCA	8.28 ± 0.69 × 10^5^	5.67 ± 0.29 × 10^7^	68.4	7.53 ± 0.38 × 10^7^
CF^*f*^→DCM	6.24 ± 1.37 × 10^6^	1.16 ± 0.27 × 10^8^	18.6	1.41 ± 0.33 × 10^8^
1,1,2-TCA^*g*^→1,2-DCA and VC	1.50 ± 0.04 × 10^6^	4.33 ± 0.05 × 10^6^	2.9	3.02 ± 0.01 × 10^7^
*–^h^*	1.39 ± 0.31 × 10^5^	5.87 ± 1.03 × 10^4^	0.4	NA^*i*^

^*a*^ Cell densities of *Dhb* were calculated from the measurements of triplicate enrichment cultures.

^*b*^ “Final” represents the time when a utilizable electron acceptor was completely depleted.

^*c*^ Fold increase in cell densities is calculated as final devided by initial.

^*d*^ Growth yields were calculated as increases in cells per μmol Cl^−^ released.

^*e*^ 59.36 μmol 1,1,1-TCA was consumed in this process.

^*f*^ 62.15 μmol CF was consumed in this process.

^*g*^ 5 μmol 1,1,2-TCA was consumed, and 1,2-DCA and VC were produced in a ratio of 1:1 in this process.

^*h*^ –, a chlorinated compound was not provided.

^*i*^ NA, yields were not calculated for insignificant growth.

### Metagenome-assembled genomes

Metagenomic sequencing was applied to investigate the microbial community compositions and functional potentials of the 1,1,1-TCA-dechlorinating enrichment culture. At the genus level, the enrichment cultures were dominated by *Dhb*, *Sedimentibacter*, *Acetobacterium*, *Propionicimonas*, and *Lentimicrobium* (Fig. S5), which is in line with the 16S rRNA gene amplicon sequencing results (Fig. 2B). A total of 105,431 contigs were assembled, with 54,766 contigs ≥500 bp, an *N*_50_ of 7,587 bp, and the largest contig of 722,444 bp. Annotation of the assembly resulted in the discovery of 91,990 putative genes, including only eight RDase genes. A total of 13 high-quality MAGs (completeness ≥90%, contamination ≤5%) and 9 medium-quality MAGs (completeness ≥50%, contamination ≤10%) were recovered by Maxbin2 (Table S3; Fig. S6).

**Fig 4:**
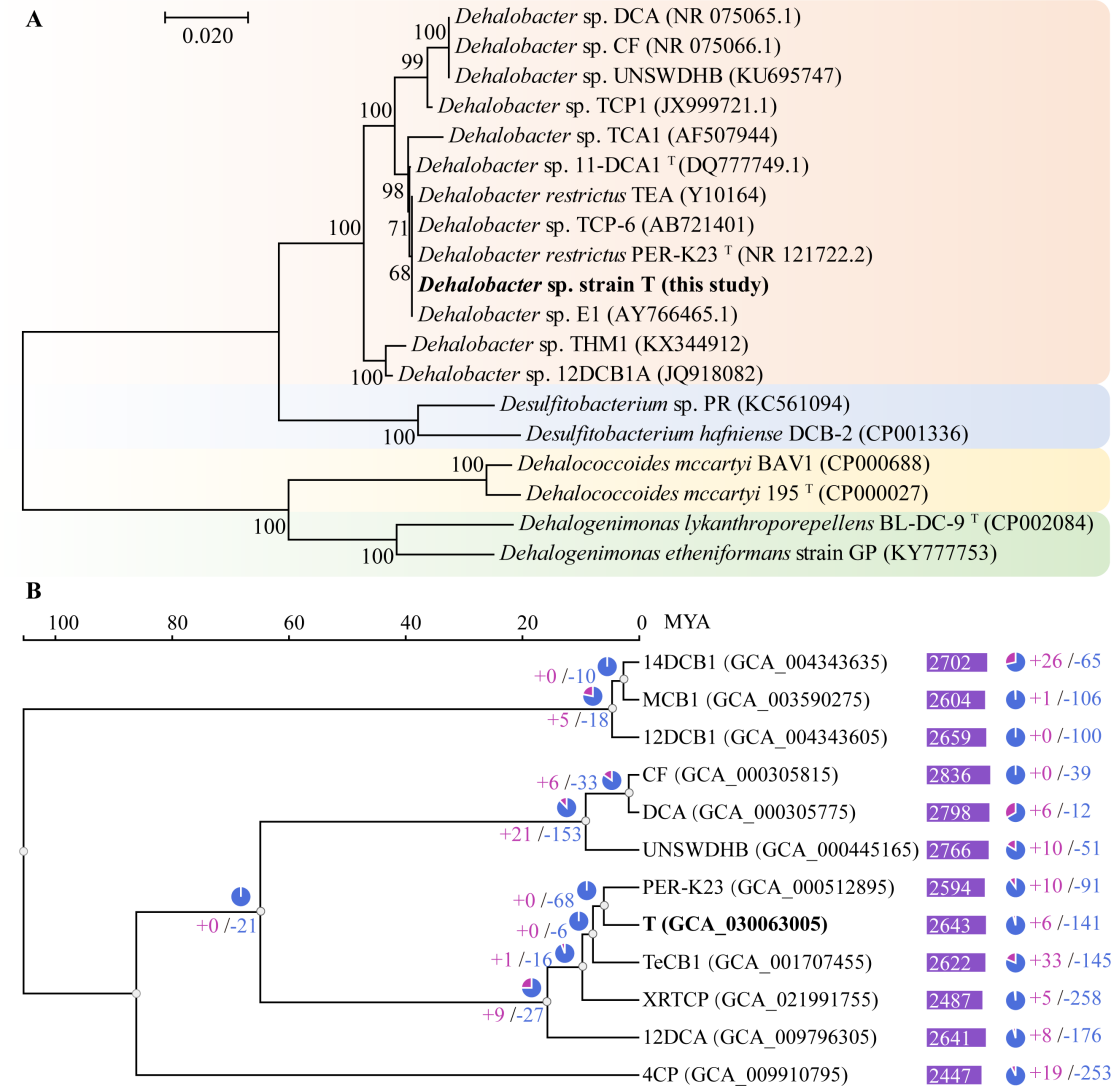
Phylogenetic affiliation of strain T to select *Dhb* strains based on 16S rRNA gene sequences (**A**). Included as outgroups are *Desulfitobacterium*, *Dhc*, and *Dhgm* 16S rRNA gene sequences. The significant bootstrap support values expressed as a percentage of 1,000 resamplings are shown at the branch points. The scale bar indicates the numbers of nucleotide differences per sequence. Gene family expansions and contractions were analyzed in 12 *Dhb* strains (**B**). This analysis involved regressing gene counts at ancestral (internal) nodes versus extant (external) nodes to assess changes in gene family size. Only statistically significant expansions and contractions (*P* value > 0.05) were considered. Red coloring indicates gene family expansions, while blue indicates contractions. GenBank accession numbers are provided in parentheses for each strain. The number displayed in the purple box represents the total number of gene families identified within the corresponding genome.

Moreover, binning of the metagenome sequences resulted in the assembly of a single high-quality MAG corresponding to *Dhb*, designated as strain T, which comprised 65 contigs with a total size of 3,006,140 bp, a G + C content of 44.5%, and an *N*_50_ value of 99,524 bp. CheckM analysis indicated that the draft genome was almost 99.9% complete with 0.3% contamination. Only one 16S rRNA gene with a full length of 1,564 bp was found in the draft genome of strain T. Phylogenetic analysis indicated that strain T is closest to *D. restrictus* strain PER-K23 with 100% 16S rRNA gene sequence similarity (Fig. 4A). The maximum likelihood phylogeny of 12 strains from the genus *Dhb* further confirmed the closest relationship between strain T and strain PER-K23 (Fig. 4B). Comparative genomic analysis of the strains revealed substantial variation in gene family distributions, with a total of 4,241 gene families identified. Among these, 400 exhibited significant expansions or contractions in their copy numbers. Notably, there was a higher prevalence of gene family contractions compared with expansions (Fig. 4B). Strain T exhibited a notably higher number of gene family contractions (141 instances) compared with strains CF (39 contractions) and UNSWDHB (51 contractions). Strain PER-K23 exhibited 91 contractions, which were more similar to strain T’s profile. These findings suggest that gene family dynamics, particularly the contraction of gene families, may play a role in the adaptive evolution and ecological niche specialization of these bacterial isolates. Further investigation into the underlying molecular mechanisms and evolutionary trajectories is warranted.

**Fig 5:**
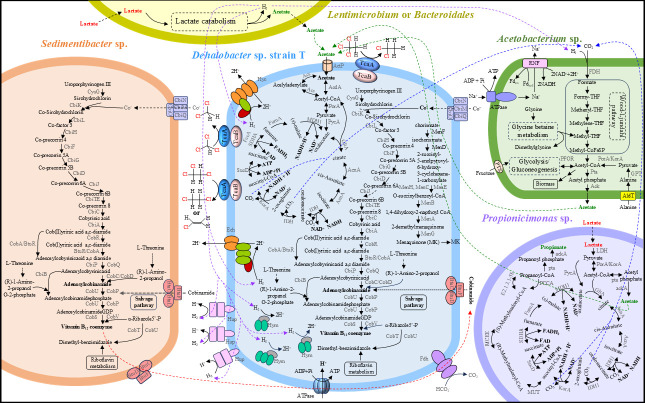
This metabolic map displays predicted pathways and interspecies metabolite transfer for strain T and its supporting populations within a 1,1,1-TCA-dechlorinating culture. Metabolic pathways were predicted from draft genomes and functional genomics analyses. Solid arrows indicate the presence of relevant genes, while dotted arrows represent unclear pathways or absent genes. Enzyme abbreviations follow KEGG annotation. Due to uncertainties in protein interactions and quinone involvement, components of the organohalide-respiring chain are shown individually in strain T.

Eight putative *rdhA* (catalytic subunit of RDase) genes were identified in the assembly of the metagenomic sequences, which were all subsequently binned into the draft genome of strain T, indicating that only a single *Dhb* population in the enrichment culture possessed functional RDases and performed reductive dechlorination. Based on the draft genome analysis, strain T lacks the complete cobalamin synthesis pathway due to the deletion of the *cbiK*, *cbiX*, and *cobR* genes involved in *de novo* corrin ring biosynthesis. This finding is consistent with the observed requirement for vitamin B_12_ during cultivation, which is essential for the function of the RDases (Fig. 5). A characteristic feature of obligate OHRB is the presence of multiple hydrogenase genes (34). Similar to strain PER-K23 (35), the draft genome of strain T is predicted to encode eight different hydrogenases, including three periplasmic membrane-bound Ni/Fe uptake hydrogenases (Hup), two six-subunit membrane-bound energy-conserving Ni/Fe hydrogenases which resemble the Hyc and Ech clusters found in *Dhc* strain 195 (36), and three Fe-only hydrogenases (Hym). In addition, one formate dehydrogenase was identified in the genome of strain T, consisting of a membrane-bound *b*-type cytochrome, a Fe/S cluster protein, and the catalytic subunit. The formate dehydrogenase in strain T is a molybdo-selenoprotein enzyme with a selenocysteine in the putative active site and shares only 35.2% similarity to the OmeA molybdoenzyme (previously annotated as formate dehydrogenase) in *Dhc* strain CBDB1, which does not contain a selenocysteine (37, 38). The presence of selenocysteine in the formate dehydrogenase may explain why *Dhb* strain TCA1 (16) can use formate as an electron donor, while *Dhc* cannot. We speculate that strain T could similarly utilize formate, although this requires further experimental verification after strain T is isolated.

### Microbial interactions

In addition to *Dhb*, several non-dechlorinating populations such as *Bacteroidales*, *Sedimentibacter*, *Propionicimonas*, *Acetobacterium*, and *Lentimicrobium* were identified in the 1,1,1-TCA-dechlorinating enrichment cultures (Fig. 2; Fig. S5). These microorganisms may be involved in providing essential carbon sources, cobalamins (39), or amino acids (40) for *Dhb*. Metagenomic analysis indicated that *Sedimentibacter*, possessing a complete cobalamin biosynthesis pathway in its draft genome (Fig. 5), could complement strain T’s inability to synthesize cobalamins. Experiments confirmed that reductive dechlorination of 1,1,1-TCA occurs in enrichment cultures without the addition of vitamin B_12_ (Fig. S3). During the lag phase preceding the onset of 1,1,1-TCA dechlorination, significant lactate transformation was observed, with lactate being entirely converted to propionate and acetate in an approximate molar ratio of 2:1 (Fig. S7). Our genomic analysis unveiled the presence of a comprehensive metabolic pathway for lactate catabolism and subsequent propionate and acetate production within the draft genome of *Propionicimonas* (Fig. 5). This finding aligns with previous reports on *Propionicimonas paludicola*, a facultatively anaerobic, propionate-producing bacterium capable of metabolizing lactate or glucose to yield propionate and acetate in a 2:1 ratio (41). Similarly, *Lentimicrobium* and *Bacteroidales*, also identified as fermenting bacteria, possess the capacity to utilize lactate for the generation of acetate and H_2_ (42). *Bacteroidales* emerged as the most abundant non-dechlorinating microorganism within our 1,1,1-TCA dechlorinating enrichment cultures (Fig. 2). This observation is consistent with previous findings that identified the dominance of the fermenting *Bacteroidales* strain CF within the ACT-3 consortium, which was shown to reductively dechlorinate 1,1,1-TCA, 1,1-DCA, and CF (43). Regrettably, the draft genome of *Lentimicrobium* and *Bacteroidales* could not be successfully binned and retrieved from the metagenome data. Furthermore, the presence of a complete Wood-Ljungdahl pathway, an acetogenic pathway enabling the fixation of CO_2_ or CO to produce acetyl-CoA and ultimately acetate (44), was identified in the draft genome of *Acetobacterium*. We hypothesize that during the lag phase preceding 1,1,1-TCA dechlorination, *Propionicimonas*, *Lentimicrobium*, and *Bacteroidales* facilitate the transformation of lactate into acetate, propionate, and H_2_. Meanwhile, *Acetobacterium* is known to have a higher H_2_ threshold (~165–346 nM) than dechlorinating bacteria such as *Dhb* (e.g., 0.06–0.3 nM) (45–47) and may compete for H_2_ to produce acetate via the fixation of CO_2_ and H_2_. Subsequently, the acetate generated and the remaining H₂ serve as the carbon source and electron donor, respectively, requisite for the growth of *Dhb* coupled with 1,1,1-TCA dechlorination. Collectively, these non-dechlorinating microorganisms likely contribute synergistically to the overall stability and functional integrity of the 1,1,1-TCA dechlorinating enrichment culture (Fig. 5). However, further investigations are required to confirm these proposed functions and the nature of their contributions to the metabolic requirements of *Dhb*.

### RDases in strain T

**Fig 6:**
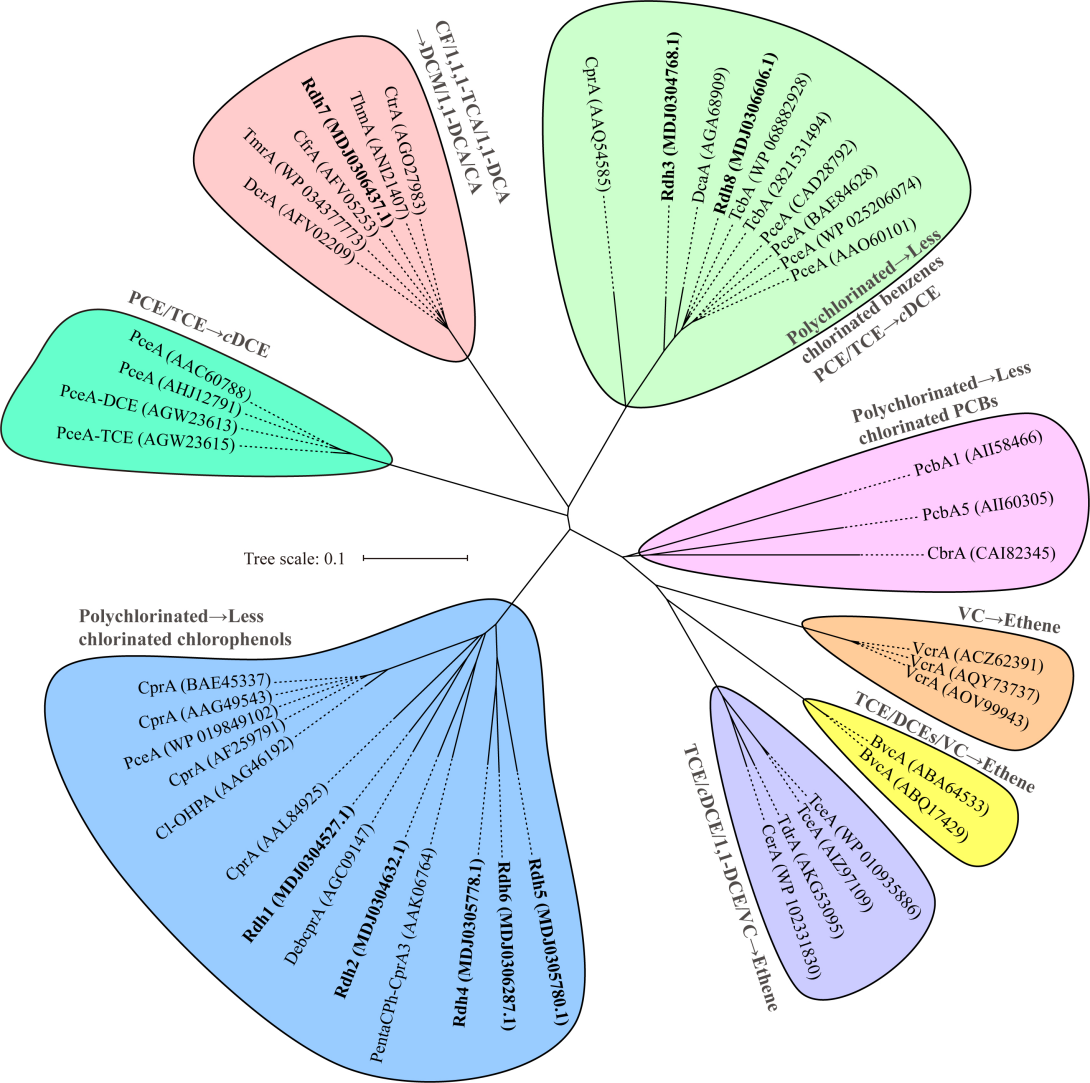
Phylogenetic analysis of 37 characterized RDases and 8 RDases (highlighted in Bold) identified in the draft genome of strain T. Detailed information on dechlorination substrates and products of the 37 characterized RDases and methods for function identification is available in Table S4. GenBank accession numbers are provided in parentheses. Branch points display significant bootstrap support values expressed as percentages derived from 1,000 resamplings. The scale bar represents the amino acid sequence divergence.

Phylogenetic analysis (Fig. 6), based on 8 RDases from strain T and 37 characterized RDases (Table S4), indicated that Rdh3 (MDJ0304768.1) in strain T shared 86% similarity with PceA (AAO60101, *Desulfitobacterium* strain PCE-S) capable of PCE dechlorination (48), while the other five RDases Rdh1, Rdh2, Rdh4, Rdh5, and Rdh6 shared 52.1–70.7% similarities with RDases such as CprA (AAL84925), DebcprA (AGC09147) (49), and PentaCPh-CprA3 (AAK06764) (50), which are involved in the dechlorination of various chlorophenols. However, despite the presence of these RDases, neither PCE nor chlorophenols were dechlorinated by the enrichment culture over an 8-month period. Rdh8 (MDJ0306606.1) shared 95.0% similarity with TcbA (WP 068882928), which is responsible for the dechlorination of 1,2,4,5-tetrachlorobenzene or 1,2,4-TCB (51). Interestingly, after 166 days, 1,2-DCB was the sole dechlorination product observed from 1,2,4-TCB when using the 1,1,1-TCA enrichment culture as the inoculum. It is notable that a putative Rdh7 (MDJ0306437.1), designated as TcaA, with a full length of 504 amino acids, shared high sequence similarity (96.7%, 94.7%, 94.3%, and 96.1%) to the four characterized 1,1,1-TCA dechlorinating RDases, CfrA, TmrA, CtrA, and ThmA (Fig. 6). The PCR assay targeting *cfrA*-like genes and the Sanger sequencing of the PCR amplicon further confirmed the presence of a *cfrA*-like RDase gene (Fig. S4B). These results indicated that TcaA of strain T may be responsible for 1,1,1-TCA-to-1,1-DCA dechlorination.

### Proteomics analysis of enrichment cultures

Proteomic analysis of the 1,1,1-TCA- and CF-dechlorinating enrichment cultures revealed 431 and 378 expressed proteins, respectively (Table S5). Notably, the putative RDase TcaA (MDJ0306437.1) and its associated membrane anchor protein TcaB (MDJ0306438.1) were detected in both cultures (Table 2). Among the identified proteins, TcaA ranked as the twelfth and sixteenth most abundant during growth with 1,1,1-TCA and CF, respectively, while TcaB exhibited the tenth and seventeenth highest expression levels following the consumption of 1,1,1-TCA and CF, respectively. Phylogenetic analyses supported the closest association of TcaA with CfrA responsible for 1,1,1-TCA and CF dechlorination (Fig. 6). Significantly, no other RdhA or RdhB proteins were observed, thereby strengthening the evidence that TcaA is the enzyme responsible for the dechlorination of both 1,1,1-TCA and CF.

**TABLE 2:** Top 15 expressed proteins identified in 1,1,1-TCA- and CF-dechlorinating enrichment cultures using the protein database of strain T^*a*^

Protein ID	Protein name	Sequence length	Razor + unique peptides	Intensity (1,1,1-TCA)	Intensity (CF)	iBAQ (1,1,1-TCA)	iBAQ (CF)	Score
MDJ0305577.1 (1/1)	Cell wall-binding repeat-containing protein	1,070	48	1.86 × 10^11^	1.46 × 10^11^	2.65 × 10^9^	2.24 × 10^9^	323.3
MDJ0305468.1 (2/2)	HU family DNA-binding protein	90	4	1.00 × 10^10^	7.22 × 10^9^	2.28 × 10^9^	1.62 × 10^9^	59.4
MDJ0304660.1 (3/7)	Pyridoxamine 5-phosphate oxidase family protein	101	8	8.22 × 10^9^	3.01 × 10^9^	8.40 × 10^8^	3.23 × 10^8^	106.7
MDJ0306955.1 (4/4)	O-acetylhomoserine aminocarboxypropyltransferase	426	21	3.43 × 10^10^	2.23 × 10^10^	7.90 × 10^8^	4.83 × 10^8^	323.3
MDJ0305517.1 (5/3)	Co-chaperone GroES	94	5	7.14 × 10^9^	5.01 × 10^9^	7.68 × 10^8^	4.95 × 10^8^	43.9
MDJ0305516.1 (6/8)	Chaperonin GroEL	544	27	2.69 × 10^10^	1.03 × 10^10^	7.00 × 10^8^	2.96 × 10^8^	323.3
MDJ0306954.1 (7/5)	Cysteine synthase A	309	16	1.22 × 10^10^	6.80 × 10^9^	5.18 × 10^8^	3.82 × 10^8^	239.3
MDJ0304850.1 (8/10)	Pyridoxamine 5-phosphate oxidase family protein	128	8	5.65 × 10^9^	2.81 × 10^9^	4.41 × 10^8^	2.23 × 10^8^	110.3
MDJ0306014.1 (9/6)	Flagellin	485	16	1.81 × 10^10^	1.08 × 10^10^	3.46 × 10^8^	3.40 × 10^8^	323.3
**MDJ0306438.1** (**10/17**)	**Reductive dehalogenase membrane anchor protein**	**105**	**2**	**7.17 × 10^8^**	**3.22 × 10^8^**	**2.39 × 10^8^**	**1.07 × 10^8^**	**13.4**
MDJ0306834.1 (11/30)	50S ribosomal protein L7/L12	125	5	1.13 × 10^9^	1.84 × 10^8^	1.84 × 10^8^	6.03 × 10^7^	37.9
**MDJ0306437.1** (**12/16**)	**Chloroform reductive dehalogenase CfrA**	**504**	**15**	**1.11 × 10^10^**	**5.87 × 10^9^**	**1.73 × 10^8^**	**1.12 × 10^8^**	**222.4**
MDJ0306841.1 (13/15)	Elongation factor Tu	400	22	7.12 × 10^9^	4.55 × 10^9^	1.57 × 10^8^	1.12 × 10^8^	323.3
MDJ0306869.1 (14/12)	30S ribosomal protein S4	209	11	2.27 × 10^9^	1.97 × 10^9^	1.50 × 10^8^	1.45 × 10^8^	90.9
MDJ0304894.1 (15/9)	Pyridoxamine 5-phosphate oxidase family protein	101	7	1.24 × 10^9^	1.85 × 10^9^	1.46 × 10^8^	2.63 × 10^8^	107.5

^*a*^ See Table S5 for the complete list. Reductive dehalogenases are indicated in bold. “Razor + unique peptides” indicates the number of peptides used for quantification. “iBAQ” is calculated as the sum of signal intensity of Razor + unique peptides divided by the number of theoretical peptides of the protein. The numbers on either side of the left oblique line in parentheses represent the ranking of the relative expression levels of *Dhb* proteins in 1,1,1-TCA- (left) and CF-dechlorinating (right) enrichment cultures, respectively.

## DISCUSSION

This study reports the successful enrichment of a novel *Dhb* strain, designated T, from river sediment in northern China. Strain T demonstrates a broad dechlorination capacity, transforming 1,1,1-TCA to 1,1-DCA, CF to DCM, 1,1,2-TCA to 1,2-DCA/VC, and 1,2,4-TCB to 1,2-DCB. The same sediment source previously yielded a diverse array of OHRB, including *Geobacter* strain IAE (52), *Dhgm* (53), and *Dhc* strain CO (54), highlighting the site’s rich OHRB biodiversity. These strains exhibit varied dechlorination capabilities, ranging from 1,2-DCA to ethene, chlorinated ethenes to ethene, and diclofenac to 2-(2-((2-chlorophenyl)amino)phenyl)acetic acid and 2-anilinophenylacetic acid. In this study, *Dhc* was initially detected in the microcosms and the second-generation enrichment culture at significant abundance (1.2–1.6%). The disappearance of 1,1-DCE produced via abiotic dehydrochlorination of 1,1,1-TCA and the concurrent detection of VC and ethene in the microcosms suggested that *Dhc* utilized 1,1-DCE, ultimately generating VC and ethene. However, following the selection for 1,1,1-TCA dechlorination in continuous transfers and minimal abiotic 1,1-DCE production, *Dhc* became undetectable and was eventually removed from the enrichment culture. This allowed *Dhb* to dominate and assume responsibility for 1,1,1-TCA dechlorination to 1,1-DCA. Prolonged incubation failed to demonstrate 1,1-DCA dechlorination by strain T. Nonetheless, the enrichment of a novel 1,1-DCA dechlorinating bacteria was achieved in newly established microcosms using the same sediment (unpublished data). Consequently, the complete dechlorination of 1,1,1-TCA to chloroethane in the Xi River sediment likely involves the sequential action of two distinct strains. While this study did not yield a novel genus capable of 1,1,1-TCA or CF dechlorination, the successful enrichment and identification of the novel *Dhb* strain T provides valuable insights into the global distribution and biogeography of *Dhb*.

### Genome comparison of strain T and other *Dhb* strains

Phylogenetic analysis based on 16S rRNA gene sequences (Fig. 4A) revealed distinct clustering patterns among the *Dhb* strains. Strain T exhibited 100% sequence similarity to strain PER-K23, while strain CF shared 100% similarity with strain DCA. However, strains T and PER-K23 shared only 92.8% similarity with strains CF and DCA, and 97.5% similarity with strain UNSWDHB. This lower similarity was attributed to the presence of an insertion sequence near the 5′ end of the 16S rRNA genes in strains CF, DCA, and UNSWDHB. Notably, after excluding these insertions, the 16S rRNA gene sequences of strains CF, DCA, and UNSWDHB were identical and differed from those of strains T and PER-K23 by only 10 nucleotides. These findings highlight the phylogenetic relationships within this group of *Dhb* strains and suggest potential evolutionary divergence driven by genetic variations, including insertions and nucleotide polymorphisms. Furthermore, comparative genomic analysis revealed that strain T and strain PER-K23 share high genome-aggregate average nucleotide identity (ANI) and digital DNA-DNA hybridization (dDDH) values (Table S6), exceeding the thresholds of 95% ANI and 70% dDDH for species demarcation (55, 56). This suggests that strain T and strain PER-K23 belong to the same species, *Dehalobacter restrictus* . In contrast, dDDH values between strain T and strains CF/DCA/UNSWDHB were below 70%, indicating significant genetic divergence. Despite their high ANI, strain T and strain PER-K23 exhibit distinct substrate ranges. Strains PER-K23 and TEA are restricted to dechlorinating PCE and TCE (6); strain E1 can respire on β-hexachlorocyclohexane and 1,2,4-TCB (39); and strain TCP-6 is capable of reductive dechlorination of various chlorinated compounds, including 2,4,6-trichlorophenol, PCE, TCE, 1,2-DCA, 2-chlorophenol, 2,4-dichlorophenol, 2,6-dichlorophenol, and 2,4,6-tribromophenol (57). Strains CF and DCA, despite their genomic similarity, also display distinct substrate ranges. Conversely, strain T shares a similar substrate range with strains CF and UNSWDHB, despite notable genomic differences. This observation underscores the complex relationship between genetic similarity and metabolic capabilities in *Dhb* strains, highlighting the importance of characterizing both genomic and phenotypic traits for understanding their ecological roles and biotechnological potential.

Phylogenetic analysis based on concatenated orthologous genomic regions (Fig. 4B) reveals a clear separation between strains CF/DCA/UNSWDHB and strains T/PER-K23. We found that gene families in strains T/PER-K23 contracted significantly compared to strains CF/DCA/UNSWDHB. While some of the eliminated duplicated family genes may be functionally redundant and potentially involved in the dechlorination of less-chlorinated metabolites, our current data do not provide direct evidence to support this hypothesis. Further investigations, including the isolation of strain T, complete genome sequencing, detailed metabolic pathway analysis, and potentially the construction of knockout mutants, are needed to determine the roles of these eliminated genes. Moreover, substantial genetic deletions were also observed in strains T and PER-K23 through genome sequence alignment. For instance, strains T and PER-K23 lack partial cobalamin biosynthesis genes, suggesting ongoing strain-specific specialization. In addition, a comparative analysis of eight metabolic pathways (i.e., glycolysis/gluconeogenesis, citric acid cycle, pentose phosphate pathway, fatty acid biosynthesis, nitrogenase, energy metabolism, Type IV pilus, and RDases) in strains CF, UNSWDHB, T, and PER-K23 (Table S7) reveals that most genes absent in strains T and PER-K23 are present in strains CF and UNSWDHB. Notably, all nitrogenase-related genes are exclusively found in strains CF and UNSWDHB. In addition, a distinctive feature of the strains CF and DCA genomes is the presence of two GC-skew arms of significantly different sizes (58), while the genomes of strains T and PER-K23 (35) exhibit two GC-skew arms of equal size. This difference in GC-skew profiles and overall gene synteny between strains CF/DCA and strains T/PER-K23 may result from potential genomic rearrangements, including insertion sequence transposition, homologous recombination, and horizontal gene transfer (58). The high ANI (>99.8%) between strains CF and DCA, after excluding large insertions, indicates recent differentiation from a common ancestor, likely driven by selective pressures on two orthologous RDase genes (*cfrA* and *dcrA*) responsible for distinct dechlorination activities (58). These findings collectively suggest that *Dhb* strains acquire organohalide respiration capabilities after their dispersal into diverse environments, leading to the evolution of distinct halogenated substrate utilization profiles. This study provides insights into the evolutionary dynamics and genomic adaptations of *Dhb* strains, highlighting the intricate interplay between genomic divergence, metabolic specialization, and ecological diversification within this genus.

### Genomic and functional diversity of 1,1,1-TCA-dechlorinating *Dhb* strains

Currently, reported strains capable of dechlorinating 1,1,1-TCA include strains TCA1 (16), CF (17), UNSWDHB (7), THM1 (19), 8M (20), and *Desulfitobacterium* sp. strain PR (18). Unlike these, the novel strain T described in this study exhibits a unique metabolic versatility, being capable of dechlorinating not only 1,1,1-TCA, CF, and 1,1,2-TCA but also 1,2,4-TCB. This expanded substrate range may be attributed to the presence of Rdh8, which shares a high sequence similarity of 95.0% with TcbA, a known RDase capable of dechlorinating 1,2,4-TCB (51). However, the observed 1,2,4-TCB dechlorination rate by strain T is relatively low, potentially necessitating long-term incubation and continuous transfers in 1,2,4-TCB-amended medium to facilitate Rdh8 adaptation and enhance dechlorination efficiency. Previous reports have documented the presence of various chlorinated benzenes, including 1,2,4-TCB, 1,4-dichlorobenzene, chlorobenzene, and 1,2,3-TCB, in the surface water overlying the sediment. This suggests that 1,2,4-TCB, or its precursors, may be present in the sediment environment, potentially serving as a selective pressure for the evolution of dechlorinating microorganisms like the *Dhb* strain enriched in this study.

To date, the RDases catalyzing 1,1,1-TCA and CF dechlorination in these strains include CtrA, TmrA, CfrA, ThmA, and TcaA, which share high amino acid sequence similarities (Fig. 6). Horizontal gene transfer appears to have played a significant role in shaping *Dhb* genomes and functions (58), with evidence suggesting that Type IV pilus-like assemblies facilitate the uptake of foreign DNA from other bacteria (59). The presence of similar 1,1,1-TCA RDases across different *Dhb* strains may, thus, be a result of HGT. Despite this similarity, subtle differences in substrate range and preference have been observed among these strains (Table S1). For instance, TcaA from strain T, CfrA from strain CF, and ThmA from strain THM1 could catalyze the transformation of CF to DCM and 1,1,1-TCA to 1,1-DCA, but not 1,1-DCA (17, 19); by comparison, CtrA of strain PR and TmrA of strains UNSWDHB and 8M can respire on all three halogenated substrates (i.e., 1,1,1-TCA, 1,1-DCA, and CF) (7, 18, 20). Although both strain UNSWDHB and strain PR can dechlorinate 1,1,1-TCA to CA, strain PR dechlorinates 1,1,1-TCA more thoroughly and demonstrates a faster dechlorination rate, while strain UNSWDHB is capable of dechlorinating higher concentrations of CF (7, 18). These subtle differences in substrate utilization may provide insights into the evolutionary mechanisms governing RDases and OHRB. Strain UNSWDHB originated from a CF-contaminated environment, whereas strain PR was isolated from a 1,1,1-TCA contaminated environment (22, 60). It is hypothesized that TmrA from strain UNSWDHB has adapted to CF-specific conditions, and the resulting partial amino acid substitutions from this adaptation may have caused 1,1,1-TCA to become a secondary substrate for TmrA (7). A recent study has implicated residues Phe80, Phe388, and Trp391 in CfrA and TmrA as determinants of isotopic fractionation of CF in strains CF and UNSWDHB (61). Notably, multiple amino acid differences exist at these positions among the 1,1,1-TCA RDases (CfrA, CtrA, TmrA, ThmA, and TcaA) (Fig. S8), suggesting that subtle variations in substrate range and preference may be influenced by substitutions at these residues in response to varying environmental and geographical conditions.

### Syntrophy between strain T and non-dechlorinating populations

Strain T exhibited slow growth in enrichment cultures supplemented with acetate and H_2_ as the carbon source and electron donor, respectively, and ceased growth after two transfers. This suggests that strain T may require additional carbon sources or nutrients not present in the medium amended with acetate and H_2_. Attempts to isolate the 1,1,1-TCA dechlorinating strain T from the enrichment culture were unsuccessful, likely due to its poor growth in isolation. Additionally, significant gene loss in strain T has resulted in incomplete metabolic pathways, such as cobalamin biosynthesis, suggesting a potential dependency on other microbes within the dechlorination community for essential nutrients. It is hypothesized that strain T and its supporting non-dechlorinating microorganisms form a stable consortium, facilitating interspecies transfer of cobalamin, amino acids, carbon sources, and electrons, thereby sustaining the organohalide respiration of strain T (Fig. 5). While the proteomics data set may offer a glimpse into the interactions between different microbial groups, we did not extensively explore these interactions in this study to maintain focus on the dechlorination process. Several challenges remain in understanding the supporting processes within the consortium. First, the enrichment cultures are complex, making it difficult to isolate specific interactions. Integrating top-down approaches (e.g., enrichment and isolation of supporting microorganisms) with bottom-up strategies (e.g., synthetic microbiomes or co-culture systems) could help elucidate these roles. Second, we analyzed samples from the final stage of the dechlorination process, which may not fully capture dynamic interactions between *Dhb* and supporting microbes over time. Our metagenomic analysis identified complete pathways for lactate catabolism and cobalamin biosynthesis in non-dechlorinating populations such as *Propionicimonas* and *Sedimentibacter*. Future studies could expand proteomic and transcriptomic analyses to examine the expression of these enzymes, offering deeper insights into the functional coordination and intricate cross-feeding interactions within the consortium and the regulatory mechanisms underlying interactions between dechlorinating and supporting microorganisms.

## Data Availability

The amplicon sequencing data are available in the Sequence Read Archive (SRA) under accession number SRR22063057. The raw metagenome sequences of the 1,1,1-TCA-dechlorinating culture were deposited in the SRA under accession number SRR22019787. The draft genome of strain T was deposited in GenBank under accession number JAPDHR000000000. The 16S rRNA gene sequence of strain T is available under accession number OP727584.

## SUPPLEMENTAL MATERIAL

Supplemental material aem.01719-24-s0001.docx Supplemental methods; Tables S1, S2, S4, and S6; and Figures S1 to S8.

Table S3 aem.01719-24-s0002.xlsx MAGs.

Table S5 aem.01719-24-s0003.xlsx Proteins.

Table S7 aem.01719-24-s0004.xlsx Genomic comparison.

ASM does not own the copyrights to Supplemental Material that may be linked to, or accessed through, an article. The authors have granted ASM a non-exclusive, world-wide license to publish the Supplemental Material files. Please contact the corresponding author directly for reuse.
