# Supplementary material for: Comparative proteogenomics reveals ecological and evolutionary insights into the organohalide-respiring Dehalobacter restrictus strain T
Source: Appl Environ Microbiol. 2025 Mar 14;91(4):e01719-24. doi: 10.1128/aem.01719-24 (PMC12016555; doi:10.1128/aem.01719-24)
Supplement: Supplemental material — Supplemental methods; Tables S1, S2, S4, and S6; and Figures S1 to S8. [file aem.01719-24-s0001.docx]

Comparative Proteogenomics Reveals Ecological and Evolutionary Insights into the Organohalide-respiring *Dehalobacter restrictus* Strain T

Xiaocui Li ^1,2^, Xiuying Li ^1^, Huijuan Jin ^1,2^, Jingjing Wang ^1^, Jun Yan ^1^, Yi Yang ^1,3*^

^1^ Key Laboratory of Pollution Ecology and Environmental Engineering, Institute of Applied Ecology, Chinese Academy of Sciences, Shenyang, Liaoning, China, 110016;

^2^ University of Chinese Academy of Sciences, Beijing, China, 100049;

^3^ CAS Key Laboratory of Forest Ecology and Silviculture, Institute of Applied Ecology, Chinese Academy of Sciences, Shenyang, Liaoning, China, 110016.

*** Corresponding authors**

Yi Yang, Institute of Applied Ecology, Chinese Academy of Sciences, 512 South Building, 72 Wenhua Road, Shenyang, Liaoning 110016, China, Phone: +86-2483970426, E-mail: yangyi@iae.ac.cn

**This supplementary file contains:** Supplementary Method; Supplementary Tables S1-S8; Supplementary Figures S1-S11.

[**Supplementary Methods**](javascript:;)

**Chemicals.** 1,1,1-TCA and CF (>99%) were purchased from XIYA REAGENT (Distributed by Linyi, Shandong, China) and CASMART (Beijing, China), respectively. Tetrachloroethylene (99%), trichloroethylene (99.9%), 1,2-dichloroethane (1,2-DCA) (99.5%), and 1,2,4-trichlorobenzene were purchased from Macklin (Shanghai, China). 1,1,2-trichloroethane (>97%) and 1,2,3-trichloropropane (>99%) were purchased from Sigma-Aldrich (St. Louis, MO). Chlorophenols (e.g., 2-chlorophenol, 3-chlorophenol, 4-chlorophenol, 2,4-[dichlorophenol](javascript:;), 2,5-[dichlorophenol](javascript:;), 3,4-[dichlorophenol](javascript:;), 2,4,6-trichlrophenol, 2,4,5-trichlorophenol) were purchased from Sigma-Aldrich (St. Louis, MO).

**Chlorinated Substrate Test.** CF (*ca*. 5 μL or 78.01 μmol, 0.85 mM aqueous concentration) containing the trichloromethyl group (-CCl_3_), chlorinated ethanes including 1,2-dichloroethane (*ca*. 6 μL or 75.98 μmol, 0.93 mM aqueous concentration) and 1,1,2-trichloroethane (*ca*. 6 μL or 64.54 μmol, 0.79 mM aqueous concentration), chlorinated ethenes including tetrachloroethylene (*ca*. 6 μL or 58.69 μmol, 0.50 mM aqueous concentration) and trichloroethylene (*ca*. 6 μL or 66.67 μmol, 0.66 mM aqueous concentration), and chlorinated phenols including 2-chlorophenol, 3-chlorophenol, 4-chlorophenol, 2,4-[dichlorophenol](javascript:;), 2,5-[dichlorophenol](javascript:;), 3,4-[dichlorophenol](javascript:;), 2,4,6-trichlrophenol, and 2,4,5-trichlorophenol (each at a concentration of 0.02 g/L) were tested using solid-free enrichment cultures amended with lactate and H_2_. All serum bottles were incubated at 30 °C in the dark without shaking for 3 months before chemical analysis.

**PCR and Quantitative Real-time PCR (qPCR) Assay**. Amplification of *Dhb*, *Dhc* and *Dhgm* 16S rRNA genes and 1,1,1-TCA-dechlorinating *cfrA*-like genes in enrichment cultures was performed on a Veriti 96-well thermal cycler (Thermo Fisher Scientific, Waltham, MA, USA) using the published primer sets (**Table S2**) with PCR mixture system and thermocycler program as previously described (1). The PCR products were checked via gel electrophoresis on a 1.5% agarose gel, and sanger sequencing was performed on an Applied Biosystems 3730XL sequencer (Thermo Fisher Scientific) by GENEWIZ Inc (Nanjing, Jiangsu, China).

To track *Dhb* cell growth during reductive dechlorination of halogenated compounds, a TaqMan chemistry**-**based qPCR assay based on the published primer pair Dhb881F and Dhb1002R and the TaqMan probe (**Table S2**) was applied for the enumeration of *Dhb* 16S rRNA genes. The qPCR was performed on a QuantStudio^TM^ 3 Real-Time PCR system (Applied Biosystems, Waltham, MA, USA). The procedures and detailed recipes of the qPCR reactions followed the published protocol (1). Plasmids carrying the partial sequence of *Dhb* 16S rRNA gene served as qPCR standards. The standard curve, ranging from 2.37 × 10^1^ to 2.37 × 10^9^ copies/reaction, showed an R^2^ value of 0.99 and an amplification efficiency of 104.18%. Cell number calculations assumed one 16S rRNA gene copy per genome for *Dhb* (2).

**Analytical Methods**. Chlorinated compounds were measured and quantified using an Agilent GC7697A headspace sampler and an Agilent GC7890B gas chromatograph equipped with a flame ionization detector (FID) and an Agilent DB-624 column (60 m × 0.32 mm × 1.8 μm; Agilent Technologies, Santa Clara, CA, USA) as previously described (1). Headspace samples taken from cultivation vessels were manually injected using a 1 ml disposable sterilized syringe (Chifeng Tianbo Medical Devices and Materials, Nei menggu, China). Liquid samples with a volume of 1 ml retrieved from the cultivation vessels were transferred to 20 ml vials and automatically injected by the Agilent GC7697A headspace sampler. The temperatures of the GC inlet and the FID were maintained at 200 °C and 300 °C, respectively. The oven temperature profile was initially held at 60 °C for 2 min, increased at 25 °C/min to 200 °C, and held isothermally for 1 min. Calibration curves were generated using 120 ml serum bottles containing 80 ml of defined mineral salt medium with known amounts of each chlorinated compound. The concentrations of chlorinated compounds were calculated by normalizing the peak areas to the standard curves.

**Phylogenetic Analyses.** Selected 16S rRNA gene sequences of *Dhc*, *Dhgm*, and *Desulfitobacterium* were retrieved from GenBank and aligned using the MEGA version 7.0.21. Protein sequences of 37 characterized RDases were retrieved from the Reductive Dehalogenase Database (<http://rdasedb.biozone.utoronto.ca>) or GenBank. The host OHRB, substrates and corresponding products, and identification method of these RDases with assigned functions were introduced in [**Table S4**](https://www.ncbi.nlm.nih.gov/pmc/articles/PMC7506110/#TS1). Phylogenetic analysis of 16S rRNA gene sequences and putative RDase proteins were performed with the maximum likelihood method using MEGA 7.0.21 software (3). The reliability of the generated trees was tested by bootstrapping with 1000 replicates. To elucidate the evolutionary relationships among the 12 *Dhb* strains, comprehensive proteome data were acquired from the NCBI repository. A maximum-likelihood phylogenetic analysis of these strains was subsequently conducted utilizing Orthovenn3, which employs the orthoMCL algorithm to infer phylogenetic orthology (4).

**Liquid Chromatography−Tandem Mass Spectrometry (LC-MS/MS) Analysis and Protein Identification in *Dhb* Genus.** After the complete dechlorination of 0.74 mM 1,1,1-TCA and 0.78 mM CF, the biomass from each individual enrichment culture, with a volume of 640 mL, was aseptically harvested via centrifugation. The collected cells were then sent to Shanghai Bioprofile Technology Company Ltd., China, for protein identification. After trypsin digestion, the peptides were desalted on C18 Cartridges (Empore™ SPE Cartridges, Sigma-Aldrich, USA) and then concentrated by vacuum centrifugation and reconstituted in 10 μL of 0.1% (v/v) formic acid (HPLC Grade, Sigma-Aldrich, USA). Mass spectrometry analysis was performed using data dependent acquisition (DDA) on a Q Exactive HF mass spectrometer, coupled with an Easy nLC system (Thermo Scientific, USA). The samples prepared for LC-MS analysis were injected into Trap Column (100 µm*20 mm, 5 µm, C18, Dr. Maisch GmbH) and then were gradient separated through chromatographic analysis column (75 µm*150 mm, 3 µm, C18, Dr. Maisch GmbH) at a flow rate of 300 nl/min. Mobile phase A was 0.1% formic acid aqueous solution, and mobile phase B is a mixture of 0.1% formic acid, 80% acetonitrile and water. The gradient was as follows: 0−2 min, 2−5% B; 2−44 min, 5−28% B; 44− 51 min, 28−40% B; 51−53 min, 40−100% B; 53−60 min, 100% B. The MS data were searched against the protein database of *Dhb* sp. strain T (Accession: ASM3006300v1) using MaxQuant software version 2.0.1.0. The following search criteria were set: enzyme specificity was trypsin; precursor mass tolerances for the main search and first search were 4.5 ppm and 20 ppm, respectively; variable modifications included methionine oxidation and N-terminal acetylation; carbamidomethylation was set as a fixed modification; the database pattern was target-reverse; and up to two missed cleavages were allowed. After retrieving the mass spectrometry data, peptide-spectrum matches (PSMs) were filtered at a false discovery rate (FDR) ≤ 0.01, and identified proteins were accepted with an FDR ≤ 0.01.

**Identification of RDases in *Dhb* sp. Strain T.** Protein sequences of 47 characterized reductive dehalogenases ([**Table S4**](https://www.ncbi.nlm.nih.gov/pmc/articles/PMC7506110/#TS1)) were retrieved from the Reductive Dehalogenase Database (http://rdasedb.biozone.utoronto.ca) (5) and were used as queries to search for putative RDases against the draft *Dhb* genome by the BLASTP program with an e-value of 1 × e^−100^ as threshold. Putative RDases were then analyzed by InterPro (https://www.ebi.ac.uk/interpro/) to assess the RDase conserved domains (e.g., twin-arginine motif, the iron–sulfur binding motif) (6).

**Functional Annotation of MAGs.** Bins were annotated with the DRAM (Distilled and Refined Annotation of Metabolism) tool (7). DRAM annotates MAGs by querying multiple databases and subsequently condenses the results to aid in the exploration of their functional and structural characteristics. It also employs functional marker genes to deduce metabolic descriptors of MAGs. For a more comprehensive understanding of DRAM's functionality, please refer to: https://github.com/WrightonLabCSU/DRAM/wiki/1.-How-DRAM-Works.

[**Supplementary Tables**](javascript:;)

**Table S1.** OHRB strains and key RDases responsible for 1,1,1-TCA and/or 1,1-DCA dechlorination.

| **Strains** | **Isolated** | **Key RdhA*^a^*** | **Length (aa)** | **Accession number** | **Dechlorination progress** | **Average chorine removal rate (mM/day)** | **Yield*^b^* (cells × 10^14^/ mole Cl^-^)** | **Reference** |
| --- | --- | --- | --- | --- | --- | --- | --- | --- |
| *Dhb* sp. strain TCA1 | Yes | *^N^* | *^N^* | *^N^* | 1,1,1-TCA→1,1-DCA→CA | 0.026 | 0.40*^c^* | (8) |
| *Dhb* sp. strain CF | No | CfrA | 456 | JX282329 | CF→DCM;  1,1,1-TCA→1,1-DCA;  1,1,2-TCA→1,2-DCA (major)/VC (minor) | 0.021 | 9.4 ± 1.7*^d^* | (6, 9) |
| *Dhb* sp. strain DCA | No | DcrA | 456 | JX282330 | 1,1-DCA→CA;  1,1,2-TCA→1,2-DCA (major)/VC (minor) | 0.022 | 24 ± 12*^e^* | (6, 9) |
| *Desulfitobacterium* sp. strain PR | Yes | CtrA | 457 | KF155162 | 1,1,1-TCA→1,1-DCA→CA;  CF→DCM (major)→MCM (minority) | 0.14 | 0.30 ± 0.13 | (10) |
| *Dhb* sp. strain UNSWDHB | Yes | TmrA | 455 | UNSWDHB_2932 | 1,1,1-TCA→1,1-DCA (incomplete, 65%);  1,1-DCA→CA (incomplete, 20%);  CF→DCM;  1,1,2-TCA→VC/1,2-DCA (1:1.7) | incomplete | incomplete | (11) |
| *Dhb* sp. strain THM1 | No | ThmA | 456 | ANI21407.1 | bromoform→dibromomethane;  CF→DCM;  1,1,1-TCA→1,1-DCA | 0.054 | ~0.57*^f^* | (12) |
| *Dhb* sp. strain 8M | No | TmrA | 455 | 40029 | 1,1,1-TCA→1,1-DCA (incomplete, 22%);  1,1-DCA→CA (incomplete, 13%);  bromoform→dibromomethane (incomplete, 48%);  CF→DCM;  1,1,2-TCA→VC/1,2-DCA (1:2.3) | incomplete | incomplete | (13) |
| *Dhb* sp. strain T | No | TcaA | 504 | MDJ0306437.1 | CF→DCM;  1,1,1-TCA→1,1-DCA;  1,1,2-TCA→VC/1,2-DCA (close to 1:1) | 0.065 | 0.75 ± 0.04*^g^* | this study |

*^a^*Key RdhA represents the RDase responsible for 1,1,1-TCA dechlorination. *^b^*Yields were calculated as increases of cells per mol Cl^−^ released. *^c d e f g^* Assuming that the genomes of corresponding *Dhb* strains have one copy of the 16S rRNA gene.

**Table S2.** Nucleotide sequences of the primers and probes used in this study.

| **Primer or probe** | **Sequence (5’-3’)** | **Purpose** | **Target gene** | **Amplicon size (bp)** | **Annealing temperature R** | **Reference** |
| --- | --- | --- | --- | --- | --- | --- |
| V3-V4-F | CCTACGGRRBGCASCAGKVRVGAAT ^a^ | PCR | Bacterial and archaeal 16S rRNA | 350 | 57℃ | (14) |
| V3-V4-R | GGACTACNVGGGTWTCTAATCC ^a^ |  |  |  |  |  |
| Dhb-254F | ACGGCTGCTAATACCGGATG | PCR | *Dhb* 16S rRNA | 1341 | 60℃ | (15) |
| Dhb-1594R | CATCTACCCCACCTTCGACG |  |  |  |  |  |
| Dhc-730F | GCGGTTTTCTAGGTTGTC | PCR | *Dhc* 16S rRNA | 621 | 55 ℃ | (16) |
| Dhc-1350R | CACCTTGCTGATATGCGG |  |  |  |  |  |
| BL-DC-142F | GTGGGGGATAACACTTCGAAAGAAGTGC | PCR | *Dhgm* 16S rRNA | 1210 | 60℃ | (17) |
| BL-DC-1351R | AACGCGCTATGCTGACACGCGT |  |  |  |  |  |
| cfrA-413 | CCCGAACCTCTAGCACTTGTAG | PCR | *cfrA* | 119 | 60℃ | (6) |
| cfrA-531 | ACGGCAAAGCTTGCACGA |  |  |  |  |  |
| Dhb-881F | CGACGCAACGCGAAGAA | qPCR | *Dhb* 16S rRNA | 122 | 61℃ | (18) |
| Dhb-1002R | CGAAGGGCACTCCCATATCTC |  |  |  |  |  |
| Dhb-probe | 6FAM-AAGGCTTGACATCCAACT-MGB ^b^ |  |  |  |  |  |

^a^ Degenerate bases, R=A/G, B=C/G/T, S=C/G, K=G/T, V=A/C/G, N=A/C/G/T, W=A/T.

^b^ 6FAM, 6-carboxyfluorescein; MGB, minor groove binder moiety.

**Table S3.** Metagenome-assembled genomes of the 1,1,1-TCA-dechlorinating culture.

**(See Table S3.xlsx)**

**Table S4.** RDases with assigned functions and their host OHRB.

| RDase (Host OHRB) | Metabolic function(s) ^a^ | | GenBank accession number | How RDase function was determined? | Reference |
| --- | --- | --- | --- | --- | --- |
|  | Substrate(s) | Product(s) |  |  |  |
| CerA (‘*Ca.* Dhgm etheniformans’ strain GP) | *c*DCE, VC | Ethene | WP_102331830 | Protein expression | (19) |
| CprA (*Desulfitobacterium chlororespirans* strain Co23) | *Ortho*-chlorophenols | Less chlorinated phenols | AAL84925 | Biochemical characterization | (20) |
| DcaA (*Desulfitobacterium dichloroeliminans* strain LMG P-21439) | 1,2-DCA | Ethene | AGA68909 | Biochemical characterization | (21) |
| CprA (*Desulfitobacterium hafniense* strain DCB-2) | Cl-OHPA | 4-HPA | AAG46192 | Biochemical characterization | (22) |
| PentaCPh-CprA3 (*Desulfitobacterium hafniense* strain PCP-1) | PCP, TeCP, TCPs | Less chlorinated phenols | AAK06764 | Transcriptional analysis | (23) |
| PceA (*Desulfitobacterium hafniense* strain PCE-S) | PCE, TCE | *c*DCE | AAO60101 | Phylogenetic inference | (24) |
| CprA (*Desulfitobacterium hafniense* strain PCP-1) | PCP, TeCP, TCP | Less chlorinated phenols | AAQ54585 | Biochemical characterization | (25) |
| PceA (*Desulfitobacterium hafniense* strain TCE1) | PCE, TCE | *c*DCE | CAD28792 | Protein expression | (26) |
| PceA (*Desulfitobacterium hafniense* strain Y51) | PCE, TCE | *c*DCE | BAE84628 | Biochemical characterization | (27) |
| CprA (*Desulfitobacterium* sp. strain PCE1) | *Ortho*-polychlorinated phenols | Less chlorinated  *ortho*-chlorophenols | AAG49543 | Biochemical characterization | (28) |
| CprA (*Desulfitobacterium* sp. strain KBC1) | Cl-HPA | HPA | **BAE45337** | Transcriptional analysis | (29) |
| PceA (*Desulfitobacterium* sp. strain PCE1) | PCE | TCE | WP_019849102 | Biochemical characterization | (28) |
| CtrA (*Desulfitobacterium* sp. strain PR) | CF/ 1,1,1-TCA,  1,1-DCA | DCM/ CA | AGO27983 | Transcriptional analysis | (30) |
| CprA (*Desulfitobacterium* sp. Viet-1) | 2,4-DCP | 4-CP | AF259791 | Phylogenetic inference | (31) |
| PceA (*Dhb restrictus* strain PER-K23) | PCE, TCE | *c*DCE | WP_025206074 | Biochemical characterization | (32) |
| CfrA (*Dhb* sp. strain CF) | CF/ 1,1,1-TCA | DCM/ 1,1-DCA | AFV05253 | Protein expression | (6) |
| DcrA (*Dhb* sp. strain DCA) | 1,1-DCA | CA | AFV02209 | Protein expression | (6) |
| ThmA (*Dhb* sp. strain THM1) | CF/ 1,1,1-TCA/ bromoform | DCM/ 1,1-DCA/ dibromomethane | ANI21407 | Proteomic analyses & biochemical characterization | (12) |
| TmrA (*Dhb* sp. strain UNSWDHB) | CF/ 1,1,2-TCA/ 1,1,1-TCA/ 1,1-DCA | DCM/ 1,2-DCA and VC/ 1,1-DCA/ CA | WP_034377773 | Biochemical characterization & Enzyme kinetic experiments | (11) |
| DebcprA (*Dhb* sp. strain TCP1) | 2,4,6-TCP | 4-CP | AGC09147 | Transcriptional analysis | (33) |
| TcbA (*Dhb* sp. strain TeCB1) | 1,2,3,4-TeCB,  1,2,4-TCB | 1,3-DCB,  1,4-DCB | WP_068882928 | Biochemical characterization | (34) |
| TceA (*Dhc* strain 195) | TCE, *c*DCE, 1,1-DCE | VC (ethene) ^b^ | WP_010935886 | Biochemical characterization | (35) |
| BvcA (*Dhc* strain BAV1) | TCE, 1,2-DCA,  DCEs, VC | Ethene | ABQ17429 | Transcriptional analysis, protein expression & Biochemical characterization | (6, 36) |
| CbrA (*Dhc* strain CBDB1) | 1,2,3,4-TeCB,  1,2,3-TCB | 1,4-DCB,1,3-DCB | CAI82345 | Protein expression | (37) |
| PcbA1 (*Dhc* strain CG1) | *Meta*-, *para*-PCBs/ PCE | Less chlorinated PCBs/ TCE | AII58466 | Transcriptional analysis | (38) |
| PcbA5 (*Dhc* strain CG5) | *Meta*-, *para*-PCBs/ PCE | Less chlorinated PCBs/ TCE | AII60305 | Transcriptional analysis | (38) |
| VcrA (*Dhc* strain VS) | DCEs, 1,2-DCA, VC | Ethene | ACZ62391 | Biochemical characterization | (39, 40) |
| VcrA (*Dhc* strain WBC-2) | VC | Ethene | AOV99943 | Phylogenetic inference | (41) |
| TdrA (*Dhgm* sp. strain WBC-2) | *t*DCE | VC | AKG53095 | Protein expression | (42) |
| BvcA (KB-1 consortium) | TCE, 1,2-DCA, DCEs, VC | Ethene | ABA64533 | Transcriptional analysis, protein expression & biochemical characterization | (43, 44) |
| TceA (KB-1 consortium) | TCE, *c*DCE | VC | AIZ97109 | Transcriptional analysis, Protein expression & Biochemical characterization | (43, 44) |
| VcrA (KB-1 consortium) | VC | Ethene | AQY73737 | Transcriptional analysis, protein expression & biochemical characterization | (43, 44) |
| PceA-DCE (*Sulfurospirillum* mixed culture SL2) | PCE, TCE | *c*DCE | AGW23613 | Enzyme assays | (45) |
| PceA-TCE (*Sulfurospirillum* mixed culture SL2) | PCE | TCE | AGW23615 | Enzyme assays | (45) |
| PceA (*Sulfurospirillum multivorans*) | PCE, TCE | *c*DCE | AHJ12791 | Biochemical characterization | (46) |
| PceA (*Sulfurospirillum multivorans* strain N) | PCE, TCE | *c*DCE | AAC60788 | Transcriptional analysis | (47) |

OHRB abbreviations: *Dhc*, *Dehalococcoides mccartyi*; *Dhgm*, *Dehalogenimonas*; *Dhb*, *Dehalobacter*

Organohalogen compound abbreviations: PCE, tetrachloroethylene; TCE, trichloroethene; *c*DCE, *cis*-dichloroethylene; *t*DCE, *trans*-dichloroethylene; 1,1-DCE, 1,1-dichloroethylene; VC, vinyl chloride; 1,2-DCA, 1,2-dichloroethane; 1,1,2-TCA; 1,1,2,-trichloroethane; 1,1,1-TCA, 1,1,1-trichloroethane; 1,1-DCA, 1,1-dichloroethane; CA, chloroethane; DCM, dichloromethane; CF, chloroform; 1,2,3-TCP, trichloropropane; 1,2-DCP, dichloropropane; Cl-OHPA, 3-chloro-4-hydroxyphenylacetate; 4-HPA, 4-hydroxyphenylacetate; 2,4,6-TCP, 2,4,6-trichlorophenol; 2,4-DCP, 2,4-dichlorophenol; PCP, pentachlorophenol; TeCP, tetrachlorophenol; TCPs, trichlorophenols; 4-CP, 4-chlorophenol; γ-HCH, γ-hexachlorocyclohexane; 1,2,3,4-TeCB, 1,2,3,4-tetrachlorobenzene; 1,2,3-TCB, 1,2,3-trichlorobenzene; 1,2,4-TCB, 1,2,4-trichlorobenzene; 1,3-DCB, 1,3-dichlorobenzene; 1,4-DCB, 1,4-dichlorobenzene; MCB, monochlorobenzene; PCB, polychlorinated biphenyl; 35-DB-4-OH, 3,5-dibromo-4-hydroxybenzoic acid; 3-B-4-OH, 3-bromo-4-hydroxybenzoic acid.

**Table S5.** All Proteins expressed in 1,1,1-TCA or CF-dechlorinating enrichment cultures using the reference protein database of *Dehalobacter* sp. strain T.

**(See Table S5.xlsx)**

**Table S6.** ANI (Average Nucleotide Identity) and dDDH (digital DNA-DNA hybridization, DDH) among the five *Dhb* genomes.

|  | **Strain T** | **Strain CF** | **Strain UNSWDHB** | **Strain PER-K23** | **Strain DCA** |
| --- | --- | --- | --- | --- | --- |
| **Strain T** | **Values below the diagonal: ANI**  **Values above the diagonal: dDDH** | 66.40 | 67.20 | 90.30 | 66.70 |
| **Strain CF** | 96.27 |  | 91.90 | 64.60 | 99.30 |
| **Strain UNSWDHB** | 96.26 | 99.24 |  | 63.20 | 91.70 |
| **Strain PER-K23** | 99.20 | 96.02 | 95.75 |  | 63.90 |
| **Strain DCA** | 96.21 | 99.68 | 99.25 | 96.38 |  |

**Table S7.** Genetic comparison of metabolic pathways among five genomes.

**(See Table S7.xlsx)**

[**Supplementary Figures**](javascript:;)


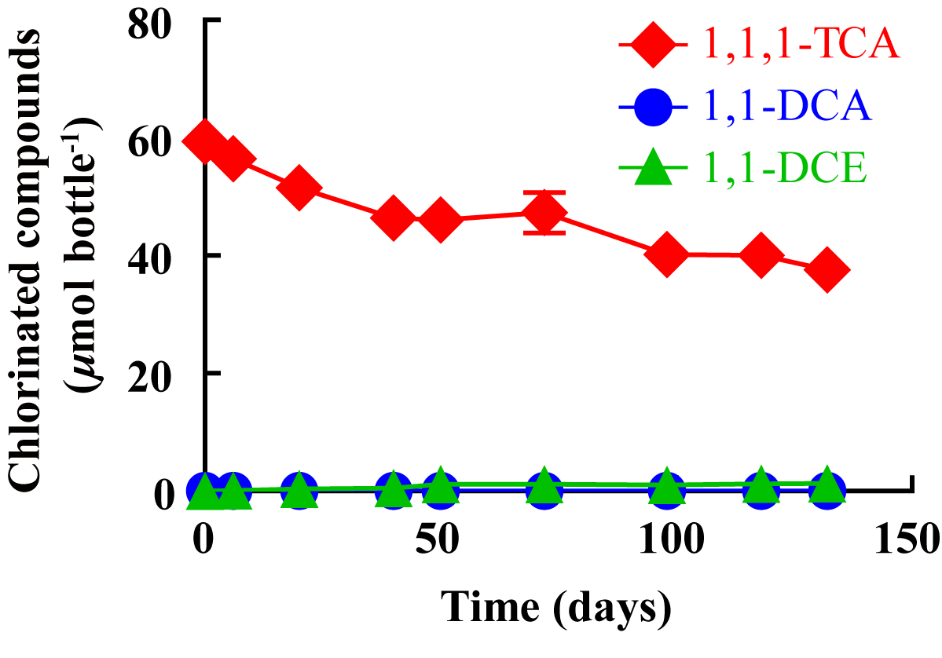


**Figure S1.** No dechlorination of 1,1,1-TCA to 1,1-DCA occurred in heat-killed enrichment culture. The error bars represent standard deviations of triplicate bottles and are not displayed when smaller than the symbol.


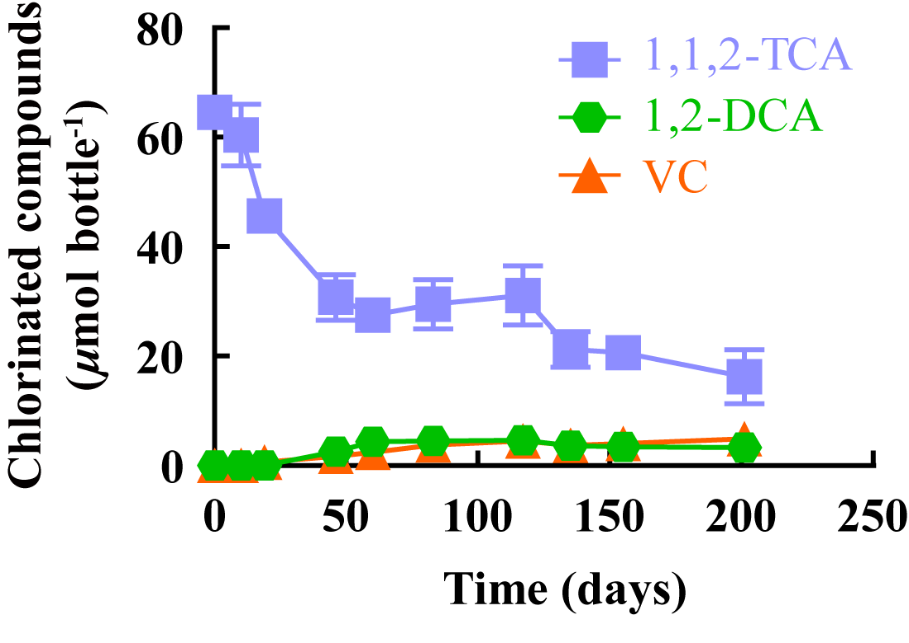


**Figure S2.** Reductive dechlorination of 1,1,2-TCA to 1,2-DCA/VC in enrichment cultures. The error bars represent standard deviations of triplicate bottles and are not displayed when smaller than the symbol.


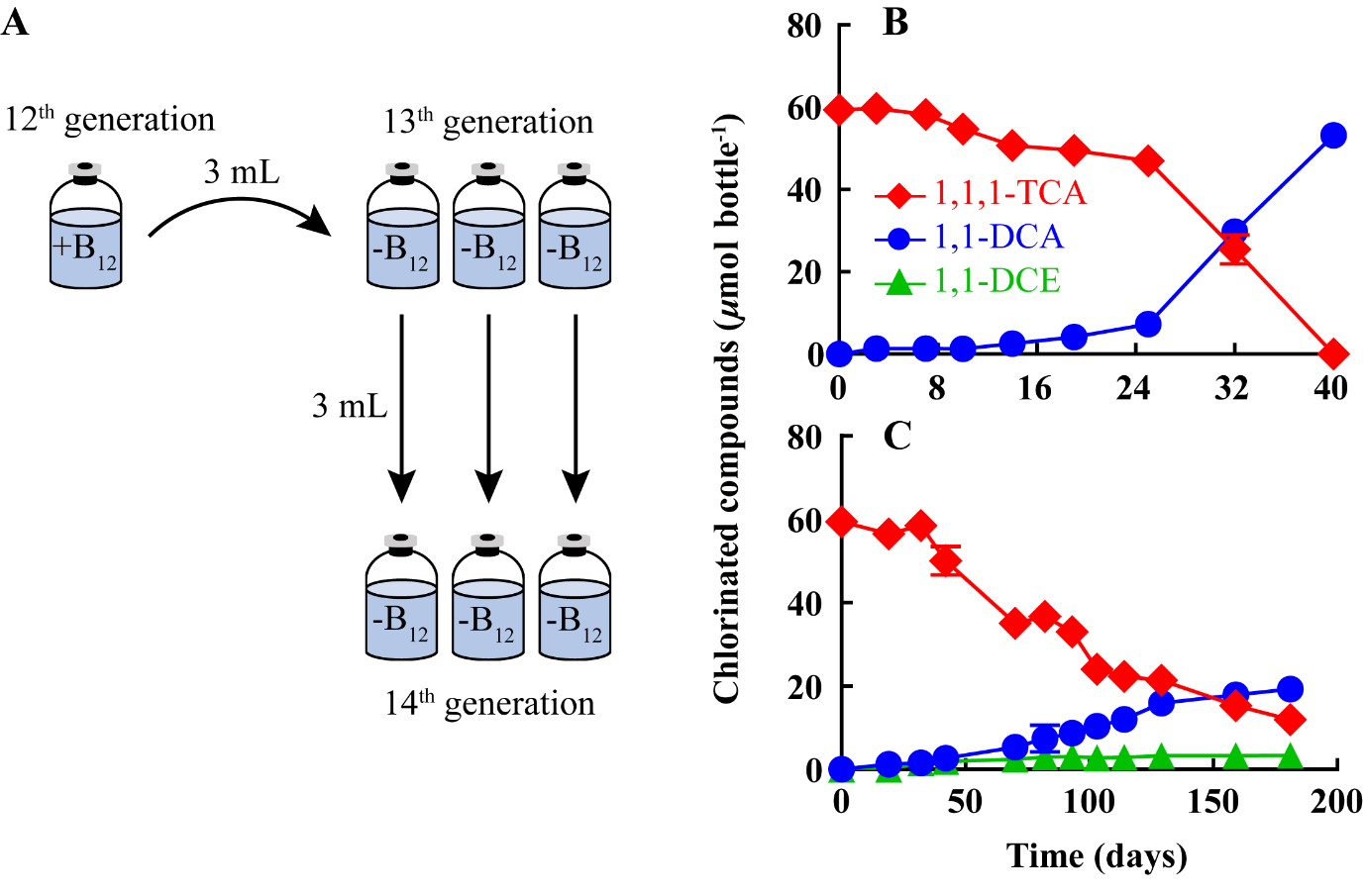


**Figure S3.** Reductive dechlorination of 1,1,1-TCA in enrichment cultures without B_12_ addition. (A) Schematic diagram of continuous transfers of enrichment cultures without B_12_ addition. (B, C) Dechlorination of 1,1,1-TCA in the 13^th^ and 14^th^ generation cultures without B_12_ addition. The error bars represent standard deviations of triplicate bottles and are not displayed when smaller than the symbol.


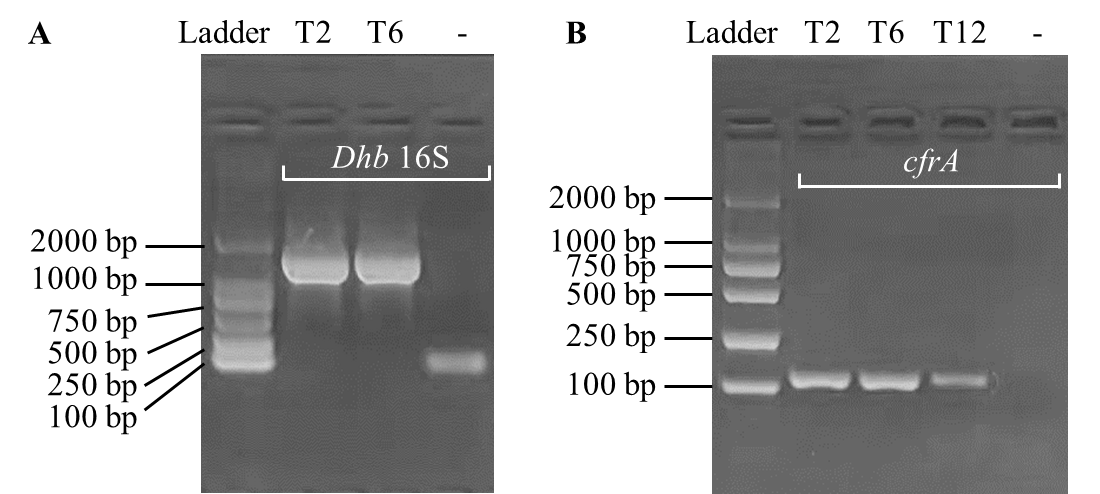


**Figure S4.** Gel electrophoresis of PCR amplicons (*Dhb* 16S rRNA gene (A) and partial *cfrA* gene (B)) generated using template DNA extracted from the 2nd (T2), 6th (T6), and 12th (T12) generation cultures. ‘-’ indicates the negative control using DNA-free water instead of DNA.


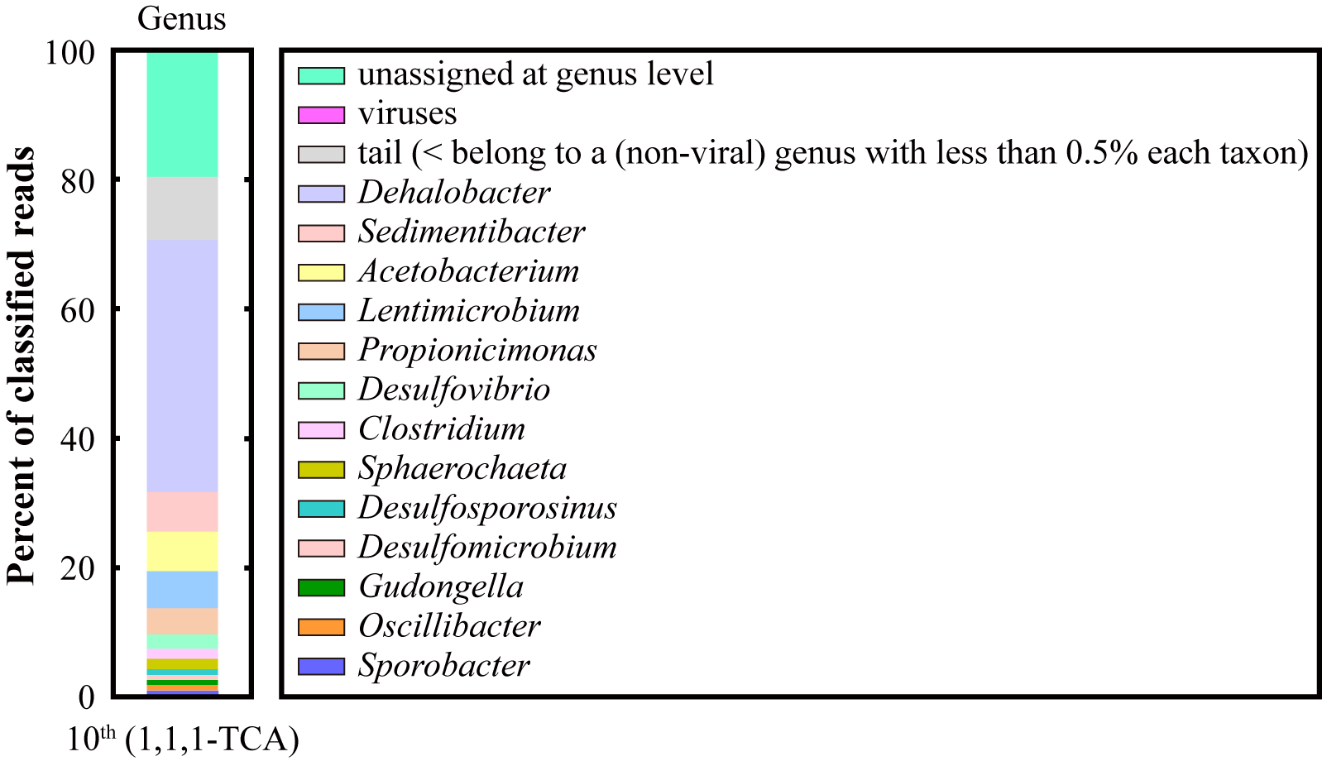


**Figure S5.** Microbial community structures at the genus level of the 10th generation culture by profiling metagenomic sequences against the NCBI nr database using Kaiju v1.7.3.


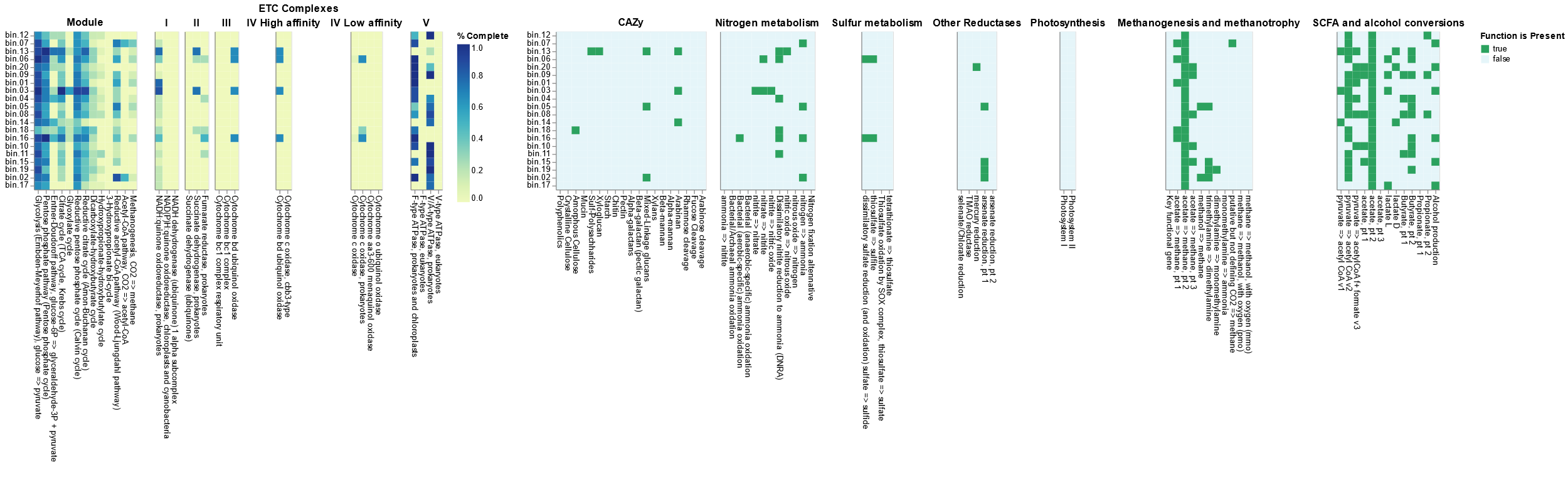


**Figure S6.** Functional annotation of metagenome-assembled genomes (MAGs) using DRAM.


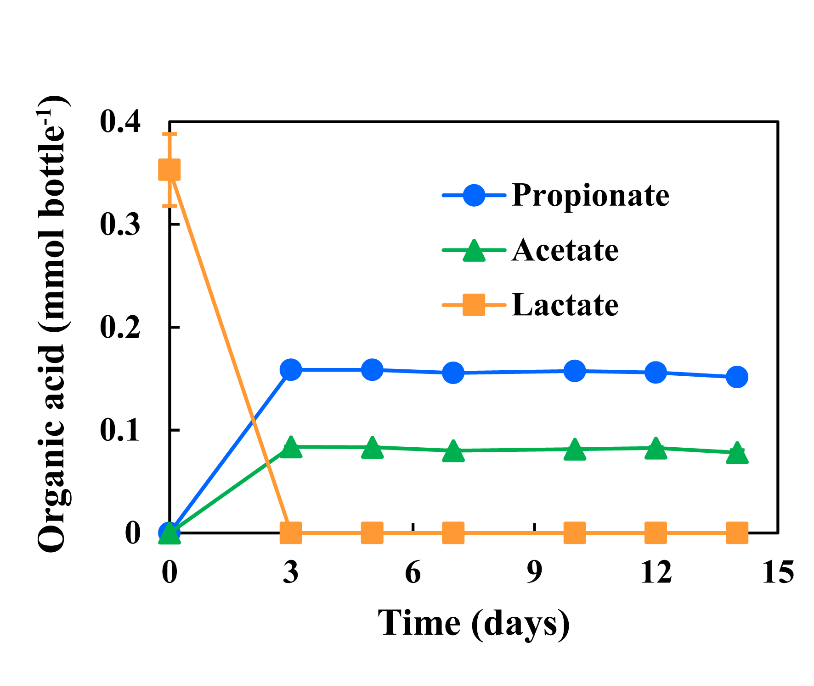


**Figure S7.** Lactate fermentation to acetate and propionate in 1,1,1-TCA-dechlorinating enrichment cultures.


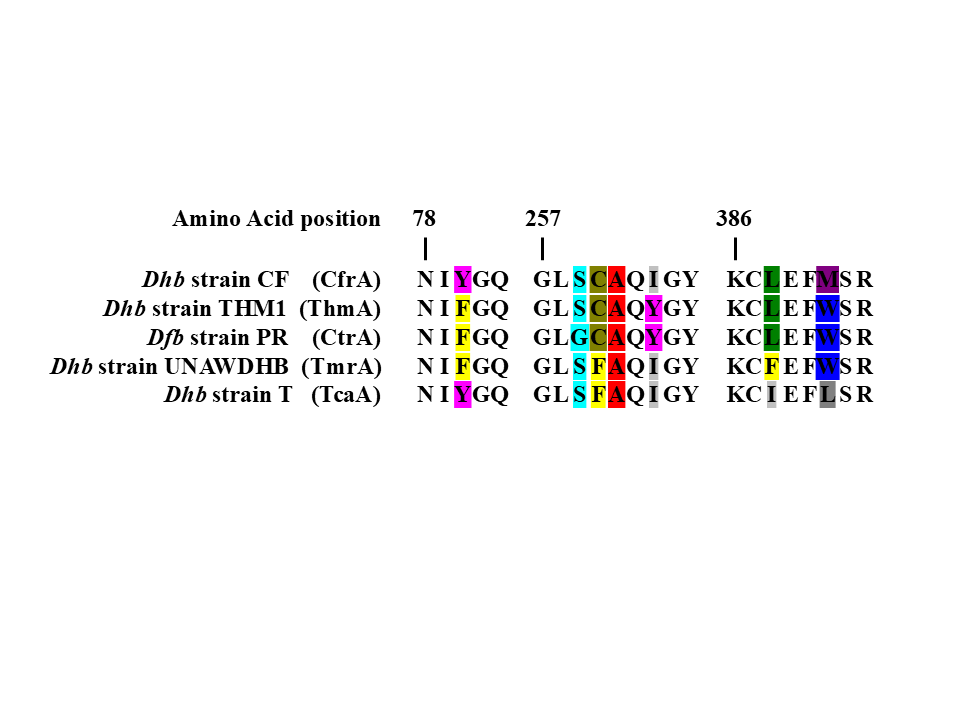


**Figure S8.** Alignment of the RdhA region, proposed to determine isotopic fractionation of CF in different OHRBs.(48) Highlighted residues represent differences from the consensus sequence. RdhAs responsible for 1,1,1-TCA transformation are shown in parentheses (*Dfb*, *Desulfitobacterium*; *Dhb*, *Dehalobacter*).

**Reference**

1. Jiang L, Yang Y, Jin H, Wang H, Swift CM, Xie Y, Schubert T, Löffler FE, Yan J. 2022. *Geobacter* sp. strain IAE dihaloeliminates 1,1,2-trichloroethane and 1,2-dichloroethane. *Environ Sci Technol* 56:3430-3440.

2. Tang S, Wang PH, Higgins SA, Löffler FE, Edwards EA. 2016. Sister *Dehalobacter* genomes reveal specialization in organohalide respiration and recent strain differentiation likely driven by chlorinated substrates. *Front Microbiol* 7:100.

3. Kumar S, Stecher G, Tamura K. 2016. MEGA7: molecular evolutionary genetics analysis version 7.0 for bigger datasets. *Mol Biol Evol* 33:1870-1874.

4. Sun J, Lu F, Luo Y, Bie L, Xu L, Wang Y. 2023. OrthoVenn3: an integrated platform for exploring and visualizing orthologous data across genomes. *Nucleic Acids Res* 51:W397-w403.

5. Molenda O, Puentes Jácome LA, Cao X, Nesbø CL, Tang S, Morson N, Patron J, Lomheim L, Wishart DS, Edwards EA. 2020. Insights into origins and function of the unexplored majority of the reductive dehalogenase gene family as a result of genome assembly and ortholog group classification. *Environmental Science: Processes & Impacts* 22:663-678.

6. Tang S, Edwards EA. 2013. Identification of *Dehalobacter* reductive dehalogenases that catalyse dechlorination of chloroform, 1,1,1-trichloroethane and 1,1-dichloroethane. *Philos Trans R Soc Lond B Biol Sci* 368:20120318.

7. Shaffer M, Borton MA, McGivern BB, Zayed AA, La Rosa SL, Solden LM, Liu P, Narrowe AB, Rodríguez-Ramos J, Bolduc B, Gazitúa MC, Daly RA, Smith GJ, Vik DR, Pope PB, Sullivan MB, Roux S, Wrighton KC. 2020. DRAM for distilling microbial metabolism to automate the curation of microbiome function. *Nucleic Acids Res* 48:8883-8900.

8. Sun B, Griffin BM, Ayala-del-Río HL, Hashsham SA, Tiedje JM. 2002. Microbial dehalorespiration with 1,1,1-trichloroethane. *Science* 298:1023-1025.

9. Grostern A, Edwards EA. 2006. A 1,1,1-trichloroethane-degrading anaerobic mixed microbial culture enhances biotransformation of mixtures of chlorinated ethenes and ethanes. *Appl Environ Microbiol* 72:7849-7856.

10. Ding C, Zhao S, He J. 2014. A *Desulfitobacterium* sp. strain PR reductively dechlorinates both 1,1,1-trichloroethane and chloroform. *Environ Microbiol* 16:3387-3397.

11. Wong YK, Holland SI, Ertan H, Manefield M, Lee M. 2016. Isolation and characterization of *Dehalobacter* sp. strain UNSWDHB capable of chloroform and chlorinated ethane respiration. *Environ Microbiol* 18:3092-3105.

12. Zhao S, Rogers MJ, He J. 2017. Microbial reductive dehalogenation of trihalomethanes by a *Dehalobacter*-containing co-culture. *Appl Microbiol Biotechnol* 101:5481-5492.

13. Soder-Walz JM, Torrento C, Algora C, Wasmund K, Cortes P, Soler A, Vicent T, Rosell M, Marco-Urrea E. 2022. Trichloromethane dechlorination by a novel *Dehalobacter* sp. strain 8M reveals a third contrasting C and Cl isotope fractionation pattern within this genus. *Sci Total Environ* 813:152659.

14. Chai B, Li X, Liu H, Lu G, Dang Z, Yin H. 2020. Bacterial communities on soil microplastic at Guiyu, an E-Waste dismantling zone of China. *Ecotoxicol Environ Saf* 195:110521.

15. Puentes Jácome LA, Edwards EA. 2017. A switch of chlorinated substrate causes emergence of a previously undetected native *Dehalobacter* population in an established Dehalococcoides-dominated chloroethene-dechlorinating enrichment culture. *FEMS Microbiol Ecol* 93.

16. He J, Ritalahti KM, Aiello MR, Löffler FE. 2003. Complete detoxification of vinyl chloride by an anaerobic enrichment culture and identification of the reductively dechlorinating population as a *Dehalococcoides* species. *Appl Environ Microbiol* 69:996-1003.

17. Chen J, Bowman KS, Rainey FA, Moe WM. 2014. Reassessment of PCR primers targeting 16S rRNA genes of the organohalide-respiring genus *Dehalogenimonas*. *Biodegradation* 25:747-756.

18. Justicia-Leon SD, Higgins S, Mack EE, Griffiths DR, Tang S, Edwards EA, Löffler FE. 2014. Bioaugmentation with distinct *Dehalobacter* strains achieves chloroform detoxification in microcosms. *Environ Sci Technol* 48:1851-1858.

19. Yang Y, Higgins SA, Yan J, Şimşir B, Chourey K, Iyer R, Hettich RL, Baldwin B, Ogles DM, Löffler FE. 2017. Grape pomace compost harbors organohalide-respiring *Dehalogenimonas* species with novel reductive dehalogenase genes. *ISME J* 11:2767-2780.

20. Löffler FE, Sanford RA, Tiedje JM. 1996. Initial characterization of a reductive dehalogenase from *Desulfitobacterium chlororespirans* Co23. *Appl Environ Microbiol* 62:3809-3813.

21. Low A, Zhao S, Rogers MJ, Zemb O, Lee M, He J, Manefield M. 2019. Isolation, characterization and bioaugmentation of an acidotolerant 1,2-dichloroethane respiring *Desulfitobacterium* species from a low pH aquifer. *FEMS Microbiol Ecol* 95:fiz055.

22. Kim S-H, Harzman C, Davis JK, Hutcheson R, Broderick JB, Marsh TL, Tiedje JM. 2012. Genome sequence of *Desulfitobacterium hafniense* DCB-2, a Gram-positive anaerobe capable of dehalogenation and metal reduction. *BMC Microbiol* 12:21.

23. Bisaillon A, Beaudet R, Lépine F, Villemur R. 2011. Quantitative analysis of the relative transcript levels of four chlorophenol reductive dehalogenase genes in *Desulfitobacterium hafniense* PCP-1 exposed to chlorophenols. *Appl Environ Microbiol* 77:6261-6264.

24. Miller E, Wohlfarth G, Diekert G. 1998. Purification and characterization of the tetrachloroethene reductive dehalogenase of strain PCE-S. *Arch Microbiol* 169:497-502.

25. Thibodeau J, Gauthier A, Duguay M, Villemur R, Lépine F, Juteau P, Beaudet R. 2004. Purification, cloning, and sequencing of a 3,5-dichlorophenol reductive dehalogenase from *Desulfitobacterium frappieri* PCP-1. *Appl Environ Microbiol* 70:4532-4537.

26. Maillard J, Regeard C, Holliger C. 2005. Isolation and characterization of Tn-Dha1, a transposon containing the tetrachloroethene reductive dehalogenase of *Desulfitobacterium hafniense* strain TCE1. *Environ Microbiol* 7:107-117.

27. Nonaka H, Keresztes G, Shinoda Y, Ikenaga Y, Abe M, Naito K, Inatomi K, Furukawa K, Inui M, Yukawa H. 2006. Complete genome sequence of the dehalorespiring bacterium *Desulfitobacterium hafniense* Y51 and comparison with *Dehalococcoides ethenogenes* 195. *J Bacteriol* 188:2262-2274.

28. Gerritse J, Renard V, Pedro Gomes TM, Lawson PA, Collins MD, Gottschal JC. 1996. *Desulfitobacterium* sp. strain PCE1, an anaerobic bacterium that can grow by reductive dechlorination of tetrachloroethene or *ortho*-chlorinated phenols. *Arch Microbiol* 165:132-140.

29. Tsukagoshi N, Ezaki S, Uenaka T, Suzuki N, Kurane R. 2006. Isolation and transcriptional analysis of novel tetrachloroethene reductive dehalogenase gene from *Desulfitobacterium* sp. strain KBC1. *Appl Microbiol Biotechnol* 69:543-553.

30. Zhao S, Ding C, He J. 2015. Detoxification of 1,1,2-trichloroethane to ethene by *Desulfitobacterium* and identification of its functional reductase gene. *PloS one* 10:e0119507.

31. Tront JM, Amos BK, Löffler FE, Saunders FM. 2006. Activity of *Desulfitobacterium* sp. strain Viet1 demonstrates bioavailability of 2,4-dichlorophenol previously sequestered by the aquatic plant Lemna minor. *Environ Sci Technol* 40:529-535.

32. Schumacher W, Holliger C, Zehnder AJ, Hagen WR. 1997. Redox chemistry of cobalamin and iron-sulfur cofactors in the tetrachloroethene reductase of *Dehalobacter restrictus*. *FEBS Lett* 409:421-425.

33. Wang S, Zhang W, Yang KL, He J. 2014. Isolation and characterization of a novel *Dehalobacter* species strain TCP1 that reductively dechlorinates 2,4,6-trichlorophenol. Biodegradation 25:313-323.

34. Alfán-Guzmán R, Ertan H, Manefield M, Lee M. 2017. Isolation and characterization of *Dehalobacter* sp. strain TeCB1 including identification of TcbA: a novel tetra- and trichlorobenzene reductive dehalogenase. *Front Microbiol* 8:558.

35. Magnuson JK, Romine MF, Burris DR, Kingsley MT. 2000. Trichloroethene reductive dehalogenase from *Dehalococcoides ethenogenes*: sequence of *tceA* and substrate range characterization. *Appl Environ Microbiol* 66:5141-5147.

36. Krajmalnik-Brown R, Hölscher T, Thomson IN, Saunders FM, Ritalahti KM, Löffler FE. 2004. Genetic identification of a putative vinyl chloride reductase in *Dehalococcoides* sp. strain BAV1. *Appl Environ Microbiol* 70:6347-6351.

37. Kube M, Beck A, Zinder SH, Kuhl H, Reinhardt R, Adrian L. 2005. Genome sequence of the chlorinated compound–respiring bacterium *Dehalococcoides* species strain CBDB1. *Nat Biotechnol* 23:1269-1273.

38. Wang S, Chng KR, Wilm A, Zhao S, Yang K-L, Nagarajan N, He J. 2014. Genomic characterization of three unique *Dehalococcoides* that respire on persistent polychlorinated biphenyls. *Proc Natl Acad Sci USA* 111:12103-12108.

39. Müller JA, B.M. R, von Abendroth G, Meshulam-Simon G, McCarty PL, Spormann AM. 2004. Molecular identification of the catabolic vinyl chloride reductase from *Dehalococcoides* sp. strain VS and its environmental distribution. *Appl Environ Microbiol* 70:4880-4888.

40. Parthasarathy A, Stich TA, Lohner ST, Lesnefsky A, Britt RD, Spormann AM. 2015. Biochemical and EPR-spectroscopic investigation into heterologously expressed vinyl chloride reductive dehalogenase (VcrA) from *Dehalococcoides mccartyi* strain VS. *J Am Chem Soc* 137:3525-3532.

41. Molenda O, Tang S, Edwards EA. 2016. Complete genome sequence of *Dehalococcoides mccartyi* strain WBC-2, capable of anaerobic reductive dechlorination of vinyl chloride. *Genome Announc* 4.

42. Molenda O, Quaile AT, Edwards EA. 2016. *Dehalogenimonas* sp. strain WBC-2 genome and identification of its *trans*-dichloroethene reductive dehalogenase, TdrA. *Appl Environ Microbiol* 82:40-50.

43. Heavner GLW, Mansfeldt CB, Debs GE, Hellerstedt ST, Rowe AR, Richardson RE. 2018. Biomarkers' responses to reductive dechlorination rates and oxygen stress in bioaugmentation culture KB-1(TM). *Microorganisms* 6.

44. Tang S, Chan WW, Fletcher KE, Seifert J, Liang X, Löffler FE, Edwards EA, Adrian L. 2013. Functional characterization of reductive dehalogenases by using blue native polyacrylamide gel electrophoresis. *Appl Environ Microbiol* 79:974-981.

45. Buttet GF, Holliger C, Maillard J. 2013. Functional genotyping of *Sulfurospirillum* spp. in mixed cultures allowed the identification of a new tetrachloroethene reductive dehalogenase. *Appl Environ Microbiol* 79:6941-6947.

46. Goris T, Schubert T, Gadkari J, Wubet T, Tarkka M, Buscot F, Adrian L, Diekert G. 2014. Insights into organohalide respiration and the versatile catabolism of *Sulfurospirillum multivorans* gained from comparative genomics and physiological studies. *Environ Microbiol* 16:3562-3580.

47. Neumann A, Wohlfarth G, Diekert G. 1998. Tetrachloroethene dehalogenase from *Dehalospirillum multivorans*: cloning, sequencing of the encoding genes, and expression of the pceA gene in *Escherichia coli*. *J Bacteriol* 180:4140-4145.

48. Phillips E, Bulka O, Picott K, Kümmel S, Edwards EA, Nijenhuis I, Gehre M, Dworatzek S, Webb J, Sherwood Lollar B. 2022. Investigation of active site amino acid influence on carbon and chlorine isotope fractionation during reductive dechlorination. *FEMS Microbiol Ecol* 98.
